# In-Depth Comparison of Adeno-Associated Virus Containing Fractions after CsCl Ultracentrifugation Gradient Separation

**DOI:** 10.3390/v16081235

**Published:** 2024-07-31

**Authors:** Mojca Janc, Kaja Zevnik, Ana Dolinar, Tjaša Jakomin, Maja Štalekar, Katarina Bačnik, Denis Kutnjak, Magda Tušek Žnidarič, Lorena Zentilin, Dmitrii Fedorov, David Dobnik

**Affiliations:** 1 National Institute of Biology, Večna pot 121, 1000 Ljubljana, Sloveniadavid.dobnik@nib.si (D.D.); 2 Jožef Stefan International Postgraduate School, Jamova cesta 39, 1000 Ljubljana, Slovenia; 3 International Center for Genetic Engineering and Biotechnology, Area Science Park, Padriciano 99, 34149 Trieste, Italy; 4 Department of Bioproducts and Biosystems, Aalto University, P.O. Box 16100, 00076 Aalto, Finland; 5 Center of Excellence in Life-Inspired Hybrid Materials (LIBER) Aalto University, P.O. Box 16100, 00076 Aalto, Finland; 6 Niba Labs d.o.o., Litostrojska cesta 52, 1000 Ljubljana, Slovenia

**Keywords:** recombinant adeno-associated viruses (rAAVs), CsCl ultracentrifugation gradient, analytical methods, digital droplet PCR (ddPCR), transmission electron microscopy (TEM), analytical ultracentrifugation (AUC), size-exclusion chromatography coupled with multi-angle light scattering (SEC-MALS), Illumina sequencing

## Abstract

Recombinant adeno-associated viruses (rAAVs) play a pivotal role in the treatment of genetic diseases. However, current production and purification processes yield AAV-based preparations that often contain unwanted empty, partially filled or damaged viral particles and impurities, including residual host cell DNA and proteins, plasmid DNA, and viral aggregates. To precisely understand the composition of AAV preparations, we systematically compared four different single-stranded AAV (ssAAV) and self-complementary (scAAV) fractions extracted from the CsCl ultracentrifugation gradient using established methods (transduction efficiency, analytical ultracentrifugation (AUC), quantitative and digital droplet PCR (qPCR and ddPCR), transmission electron microscopy (TEM) and enzyme-linked immunosorbent assay (ELISA)) alongside newer techniques (multiplex ddPCR, multi-angle light-scattering coupled to size-exclusion chromatography (SEC-MALS), multi-angle dynamic light scattering (MADLS), and high-throughput sequencing (HTS)). Suboptimal particle separation within the fractions resulted in unexpectedly similar infectivity levels. No single technique could simultaneously provide comprehensive insights in the presence of both bioactive particles and contaminants. Notably, multiplex ddPCR revealed distinct vector genome fragmentation patterns, differing between ssAAV and scAAV. This highlights the urgent need for innovative analytical and production approaches to optimize AAV vector production and enhance therapeutic outcomes.

## 1. Introduction

Adeno-associated virus (AAV) is a small, non-enveloped virus of the *Parvoviridae* family. Due to their low immunogenicity, robust gene expression, and replication defectiveness, recombinant AAVs (rAAVs) are widely used in gene therapies, with more than 100 on-going or completed clinical trials [1]. Despite numerous clinical trials, only a few of them have received European Medicines Agency (EMA) and Food and Drug Administration (FDA) approval so far. As of April 2024, there are five AAV-based medicines approved by the EMA (Luxturna, Zolgensma, Upstaza, Roctavian, and Hemgenix) and five approved by the FDA (Luxturna, Zolgensma, Roctavian, Hemgenix, and Elevidys) [2]. A recent review of the clinical trials involving rAAVs pointed out that the optimal dosing regimen remains elusive, even after more than two decades of research [1]; however, this is tied to the adequate purification of samples to eliminate impurities and to enable accurate quantification of the complete viral genomes.

Usually, HEK293 cells are used for viral particle production, and after harvest, particles are purified using gradient centrifugation or column-based chromatography [3]. Purified AAVs are then subjected to characterization using different analytical methods to evaluate their quality attributes. The content ratio (empty to full capsids) is traditionally assessed by analytical ultracentrifugation (AUC), transmission electron microscopy (TEM), qPCR/dPCR-ELISA combination and/or other novel methods that are also emerging [4,5,6,7,8,9]. AUC quantifies different AAV particle populations (empty, partially filled, full, heavy) but requires a relatively large amount of sample, which can be difficult to obtain for regular in-process testing [8,10]. TEM is the only technique that allows direct visualization of AAV capsids and also reveals impurities and aggregates, but determining the content ratio requires image analyses that are time-consuming [8], unless employing expensive automated image recognition software (e.g., Vironova Analyzer Software, VAS [11]).

Indirect measurements of the content ratio usually combine the results from two methods, one determining the capsid titer and the other quantifying the genome titer (also called the vg/vp ratio). The capsid titer can be easily determined using enzyme-linked immunosorbent assay (ELISA) or optical density methods [12,13], and for the genome titer, quantitative PCR (qPCR) and lately also digital PCR (dPCR) are used [14,15,16]. In addition, some novel methods such as size exclusion chromatography coupled with either multi-angle light scattering (SEC-MALS) [13,17], fluorescence and triple wavelength UV detection (SEC-FLD-TWUV) [18], or charge detection mass spectrometry (CDMS) [10,19] attempt to combine both measurements in one analysis. Apart from the content ratio, the identification and quantification of host cell DNA and protein impurities represent a very important aspect of AAV production. Usually, ELISA or liquid chromatography mass spectrometry (LC-MS) are used to determine protein impurities [20] and qPCR/dPCR or sequencing are used for residual DNA impurities [10,21,22].

Recent studies have compared various analytical strategies for the quantification of rAAVs’ particle content [4,6,8,10,16,23]. However, none of these studies simultaneously evaluated both single-strained (ssAAV) and self-complimentary (scAAV) vectors, nor have they thoroughly assessed the presence of impurities. Our research focused on different AAV fractions after CsCl ultracentrifugation (empty, intermediate, full, and heavy), which have shown comparable infectivity. To elucidate the reason for the similar infectivity and to explore further the differences between the samples from four fractions after ultracentrifugation, we have performed their thorough characterization, using several, and in some cases orthogonal, approaches. Here, we present the obtained results, which confirmed that the differences between the samples were not as extensive as expected based on their origin in terms of the position in the gradient.

## 2. Materials and Methods

### 2.1. AAV Production and Purification

Single-strand and self-complementary recombinant vectors expressing enhanced green fluorescent protein (GFP) were prepared in two technical replicates by the AAV Vector Unit at the International Centre for Genetic Engineering and Biotechnology Trieste(ICGEB,), as described previously [24], with a few modifications. Briefly, infectious AAV vector particles were generated in HEK293T cells cultured in roller bottles by a triple transfection, cross-packaging approach whereby the vector genome was packaged into AAV capsid serotype-9 [25]. Purification of the viral particles was performed by PEG precipitation and two subsequent CsCl gradient centrifugations [26]. After the 2nd ultracentrifugation, each ultracentrifugation tube was punctured at 1 cm from the base with a 21 g needle and four 1.5 mL fractions were collected, including the visible full and empty bands. The refractive indexes were read with a refractometer to calculate the respective densities. Based on the expected value of the full viral particles and the position in the gradient, the fractions were classified as heavy (H) (RI 1.373 for ssAAV or 1.374 for scAAV), full (F) (RI 1.370 for both vectors), intermediate (I) (RI 1.368 for ssAAV or 1.367 for scAAV) or empty (E) (RI 1.364 for both vectors). Upon receipt, the samples were aliquoted into 100 µL and stored at −80 °C. A new aliquot was used for each analysis so that all the analytical methods, even if performed at different times, were performed with a sample from the same freeze/thaw cycle. A schematic presentation of the viral vectors and fractions studied can be found in the Supplementary Materials (Figure S1).

### 2.2. Transduction Efficiency

HEK293 cells were plated at a density of 1.8 × 10^5^ cells/well in a 24-well plate. The subsequent day, the cells were infected with vector fractions at an MOI of 1 × 10^4^ vector genomes (determined with qPCR), with the addition of the proteasome inhibitor MG132 at a concentration of 2 μM to enhance the transduction. The cell medium was changed 5 h after infection. The % of GFP-positive cells was quantified 2 days after transduction using FACS analysis.

### 2.3. Transmission Electron Microscopy (TEM)

Transmission electron microscopy was used for observation of the viral particles, aggregates, and other impurities in all the examined samples, as previously described [14]. Briefly, 13 µL of each sample was added in duplicates to a piece of parafilm. Freshly glow-discharged (GD+) copper grid (400 mesh, formvar-carbon coated) and copper grid without glow discharging (GD−) were placed on the sample droplets for 5 min. The grid was removed from the sample and the excess liquid was drained by touching the edge of the grid with a piece of clean filter paper. The grid was rinsed with 3–5 drops of Milli-Q water and dried again with a piece of clean filter paper. A drop of 1% (*w*/*v*) water solution of uranyl acetate was placed on the grid and immediately blotted with a clean piece of filter paper. The grid was examined using a TEM Philips CM 100 (FEI) with an accelerating voltage of 80 kV. The overall quality of the grid was evaluated under low magnification. Negatively stained areas were selected and the integrity of the viral particles (full, partially filled, empty and damaged) and the presence of aggregates and host cell protein impurities were examined at higher magnification. The observation was repeated on at least 5 negatively stained grid squares and representative micrographs were taken using the ORIOUS SC 200 CCD camera (Gatan, Inc., Pleasanton, CA, USA) with automatic settings using Gatan Digital Micrograph software version 2.10.

The micrographs were analyzed manually. At least 2000 viral particles were counted and defined as full, empty, partially filled, and damaged for each sample studied (as previously described by our group [14]. Briefly, particles that appeared as spherical, uniformly electron-transparent structures were determined to be full. Ring-shaped structures with an electron-dense interior were classified as empty. Empty particles with visibly damaged capsids were classified as damaged. Particles with an incompletely electron-transparent interior were classified as partially filled. The average % and standard deviation (SD) of each viral particle population present were calculated in Excel (Microsoft) from all the studied micrographs for each sample. Additionally, damaged particles were excluded from calculations of the % full viral particles (when those results were directly compared to other methods, since most of them (ELISA, AUC and SEC-MALS) do not separately specify the presence of damaged particles.

### 2.4. Analytical Ultracentrifugation (AUC)

Sedimentation velocity experiments were performed on the fractions using a Beckman Coulter Optima AUC. The fractions from both batches were combined due to the high volume requirements. All the experiments were carried out at 20 °C using standard 12 mm 2-channel centerpieces in an An-60 Ti rotor. The measurements were performed in absorbance mode at a wavelength of 230 nm and two rotor speeds: 12,000 and 20,000 RPM. The concentrations of the samples were in the range of 0.5–0.8 OD at 230 nm. The reference sectors were filled with PBS buffer for all the measured samples. The raw data of 350 scans of concentration versus radial were collected. The raw data were fitted to produce c(s) distribution functions using SEDFIT software [27]. Scans 1–100 of each experiment were fitted to the c(s) model with a resolution of 200, range of 1–200 Svedberg (S), floating meniscus and frictional ratio and the maximum entropy regularization with a factor of 0.68. The root mean-square deviation (RMSD) for A230 was in the range of 0.004 to 0.005 for the samples without baseline aberrations. The samples with significant small molecule signal returned RMSD values around 0.01 to 0.02 and had limited precision and accuracy. The trapezoid rule was used to integrate the percent of signal between the sedimentation coefficient boundaries for defined regions corresponding to different relevant species. The regions were 0–25 S, the excluded low S region; 25–55 S, the macromolecular region; 55–70 S, the empty capsid region; 70–80 S, the partial capsid region; 80–120 S, the full capsid region; 120–190 S the larger than full capsid region; and greater than 190 S, the very large aggregate region. The 0–25 S region contains macromolecular species not relevant to AAV capsids and could include fragments of capsids, contaminating macromolecules, and very low S artifacts of fitting, and it was thus not included in the total signal for the calculations of the percent of AAV species. The resolution of species depends upon the signal to noise ratio, the quality of the curve fit, and the number of species present in the sample. Regularization of the c(s) distribution will in cases of high noise over-smooth the distribution and the boundaries of different regions may be adjusted by visual inspection of the distribution.

### 2.5. Batch Dynamic Light-Scattering (DLS) Measurements

Preliminary batch dynamic light-scattering (DLS) measurements of all four fractions from one ssAAV and one scAAV batch were performed using a DynaPro^®^ NanoStar (DLS/SLS detector, Wyatt Technology, Santa Barbara, CA, USA). The samples were analyzed using 4 µL of each sample in a quartz cuvette, using an acquisition time of three seconds [28,29].

### 2.6. SEC-MALS-DLS-RI Measurements

All four fractions from one ssAAV and one scAAV batch were analyzed using multi-angle light scattering (MALS) coupled with size exclusion chromatography (SEC). A system from Wyatt Technology was used, consisting of a DAWN 18-angle light-scattering detector (including a QELS module for online dynamic light-scattering measurements) and an Optilab refractive index detector. The LC system used was an Agilent 1260 Infinity II (isocratic pump, vial sampler, degasser), including UV detection at 280 and 260 nm (InfinityLab Max-Light cartridge cell G4212-6008). An SEC column from Wyatt Technology was used to separate the AAV samples (WTC-050N5; 5 µm, 500, 500Å; 4.6 mm ID × 300 mm; 30 µL injection volume, flow rate 0.3 mL/min). The separation was performed at room temperature. The determination of the critical quality attributes of the AAV samples was performed using a special AAV module for the ASTRA software 8.0.1.21 (Wyatt Technology). The calculation was based on the conjugate analysis [6,17,30,31,32].

### 2.7. Viral Genome Titer Determination

Firstly, the viral genome titer of all four fractions from one ssAAV and one scAAV batch was determined at ICGEB by qPCR using SybrGreen Technology, targeting GFP. Serial dilution of the plasmid containing the vector genome was used as a standard curve, as described previously [33]. Secondly, absolute quantification of the vector genomes was performed by droplet digital PCR (ddPCR) using two different assays targeting the CMV enhancer or GFP gene (Table 1) to cover different parts of the genome and assess the variability between different assays. The GFP assay was developed as part of this study, as previously described by our group [14]. All the ddPCR runs were performed as previously described [14]. In addition, all the samples were diluted 1:5 in dilution buffer containing PCR buffer II without MgCl_2_ (Applied Biosystems, Foster City, CA, USA), 25 mM MgCl_2_ (Applied Biosystems) and 0.05% Pluronic F-68 (Thermo Fisher Scientific, Waltham, MA, USA) prior to the assay to minimize the inter-assay variability due to possible viral particle aggregation.

Moreover, the fragmentation of the vector genomes was investigated in four fractions from one batch of ssAAV and one batch of scAAV using single dye (FAM) duplex ddPCR for the simultaneous detection of two targets (CMV and SV40). See their position on the vector genome in Figure S1. The duplex ddPCR was performed in the same manner as the simplex ddPCR. In contrast to the simplex ddPCR, the threshold tool of QuantaSoft analysis software 1.7.4 (BioRad) was used to manually apply the threshold line (TL) such that double-negative (TL at 2500), single-positive (CMV—TL at 10,000 or SV40—TL at 6500), and double-positive droplets (CMV and SV40—TL at 13000) were separated. The percentages of the full genome, SV40 fragment and CMV fragment were calculated from the ratio of the concentration of the target group to the concentration sum of the total droplets [35]. Additionally, the percent linkage value (i.e., the proportion of droplets in which both SV40 and CVM targets were present/linked on one molecule) was calculated [36].

### 2.8. Viral Capsid Titer Determination

The total number of intact viral capsids was determined in all the samples using the AAV9 titration ELISA kit (Progen Biotechnik GmbH, Heidelberg, Germany) according to the manufacturer’s instructions. To fall within the recommended testing range for ELISA, the theoretically expected number of viral capsids was determined based on the combination of the results of the vector genome titer and particle analysis by TEM. Based on the calculated theoretical values, the samples were diluted in 20× assay buffer (part of the ELISA kit). Three dilutions were tested in duplicate for each sample (the lowest dilution was tested only once). The concentration of intact viral capsids was calculated with the MyAssay Microsoft Excel Office Professional Plus 2016 add-in (MyAssays Ltd., Brighton, UK) using a 4-parameter logistic fit (4PL) curve.

### 2.9. Determination of Host Cell Impurities

Residual host cell DNA was extracted from all the samples using the PrepSEQ Residual DNA Sample Preparation Kit (Applied Biosystemsaccording to the manufacturer’s automated protocol for the MagMAX express-96 magnetic particle processor (Applied Biosystems). The extracted DNA was analyzed using the commercially available resDNASEQ Human Residual DNA Quantitation Kit (Applied Biosystems) according to the manufacturer’s instructions, with a few modifications. After centrifugation, the plate was transferred to the QuantStudio 7 Flex Real-Time PCR System (Applied Biosystems) and analyzed using the QuantStudio Real-Time PCR Software v1.3 (Applied Biosystems).

The residual host cell proteins were evaluated in all the fractions using the HEK 293 Host cell proteins ELISA kit (Cygnus Technologies, Leland, NC, USA) following the manufacturer’s instructions. The absorbance was measured using the Sunrise microplate reader (Tecan, Redwood City, CA, USA) at 450 and 650 nm. The amount of host cell proteins was calculated with Microsoft Excel using the second-order polynomial equation.

### 2.10. Multi-Angle Dynamic Light Scattering (MADLS)

A total of 20 µL of one batch of scAAV fractions was analyzed by Zetasizer Ultra (Malvern Panalytical, Malvern, UK) using quartz cuvettes. The standard parameters were used to measure the particle size, particle concentration and presence of aggregates.

### 2.11. High-Throughput Sequencing (HTS)

Due to the limited total volume of the original samples, 100 µL of each technical replicate was combined to obtain sufficient DNA for analysis. Because we wanted to compare the genetic material inside and outside the capsids, 200 µL of the sample mixture was divided in half and only one half was treated with Ambion DNase I (Thermo Fisher Scientific) before DNA extraction. Briefly, 100 µL of the sample was treated with 10 units of Ambion DNase I in the 200 µL reaction volume that also contained Ambion DNase I buffer (Thermo Fisher Scientific) and nuclease-free water (NFW). The mixture was mixed gently and incubated at 37 °C for 30 min. For samples without DNase I pretreatment, 5 µL of NFW was added to the reaction mixture instead of Ambion DNase I. To stop the DNase I pre-treatment, EDTA was added (final concentration 5 mM) and the mixture was incubated at 65 °C for 10 min. Then, 200 µL of the DNase reaction was used to extract the total DNA using the QIAamp MinElute Virus Spin Kit (Qiagen, Hilden, Germany), following the manufacturer’s protocol for purification of viral nucleic acids from plasma or serum with few modifications. The total DNA was eluted in 60 µL of NFW. The DNA concentration was determined using the Qubit dsDNA HS Assay kit (Invitrogen, Carlsbad, CA, USA) according to the manufacturer’s instructions. The length of the extracted vector genomes was evaluated using the Bioanalyzer High Sensitivity DNA Kit (Agilent Technologies, Santa Clara, CA, USA) according to the manufacturer’s instructions. Next, for one half of each sample after DNase treatment (with and without DNase), the 2nd DNA strand was synthesized in a 50 µL reaction that contained 6 µM random hexamers (Invitrogen), 2 mM dNTPs (Invitrogen), 10 U DNA polymerase I (NEB, Ipswich, MA, USA), NEB buffer 2 (NEB), NFW, and 25 µL of extracted DNA. First, the random hexamers, buffer, NFW, and sample were mixed and incubated at 95 °C for 5 min. The mixture was then cooled on ice and dNTPs and polymerase were added. The samples were then incubated at 37 °C for one hour. Randomly primed DNA synthesis was terminated by adding EDTA to a final molarity of 0.1 mM. The control for the sequencing was NFW without DNase treatment and with a synthesized 2nd DNA strand. Since almost no nucleic acids were expected in this control, which is not suitable for library preparation, λ-phage DNA (NEB) was added after the 2nd DNA strand synthesis to a final concentration of 10 ng/µL.

The library preparation and sequencing were performed by Novogene, using the Illumina NovaSeq paired-end 150 nts sequencing workflow. The raw sequencing reads were analyzed by CLC Genomics Workbench 20.0.4 (Qiagen). After quality inspection, the reads were trimmed using a quality filter (limit = 0.03; maximum 1 ambiguous nucleotide allowed) and an automatic adapter read-through and filtered by size, where reads shorter than 30 bp were removed. The trimmed and filtered reads were then mapped (length fraction = 0.95, similarity fraction = 0.95) in a sequential manner to the reference sequences: first to the reference sequence of the product (vector sequence ITR to ITR—2479 bases for ssAAV, 4534 bp for scAAV). Afterwards, the unmapped reads were mapped to the reference sequences of the plasmids used in the procedure (product backbone, pHelper and pAAV2/9n). For hcDNA identification, the remaining unmapped reads were mapped to the reference human genome sequence (homo sapiens hg38).

The raw HTS data generated in the present study are available from the NCBI Sequence Read Archive under the BioProject ID: PRJNA1102173.

### 2.12. Graphical Representation

The graphical representation was performed using Prism V10.1.0 GraphPad Software. The illustrations of the viral vectors and fractions in the Supplementary Materials were created with BioRender.com (https://app.biorender.com/ (accessed on 22 July 2024)).

## 3. Results

In the present study, we performed an in-depth comparison of four AAV-containing fractions from the second CsCl ultracentrifugation gradient. Conventional ssAAV and scAAV vectors, both expressing GFP, were prepared, purified, and characterized side by side. Two technical replicates of each viral vector were included in the study.

### 3.1. CsCl Extracted Fractions Showed Unexpectedly Similar Transduction Efficiencies

First, we compared the infectivity of the extracted fractions (Table 2). According to the initial qPCR, the highest level of infectivity was expected in the full fractions (RI 1.370), but the observed infectivity levels were comparable in all the fractions of each viral vectors.

### 3.2. Different Viral Particle Populations Were Identified by TEM and AUC in All Fractions

**Figure 1:**
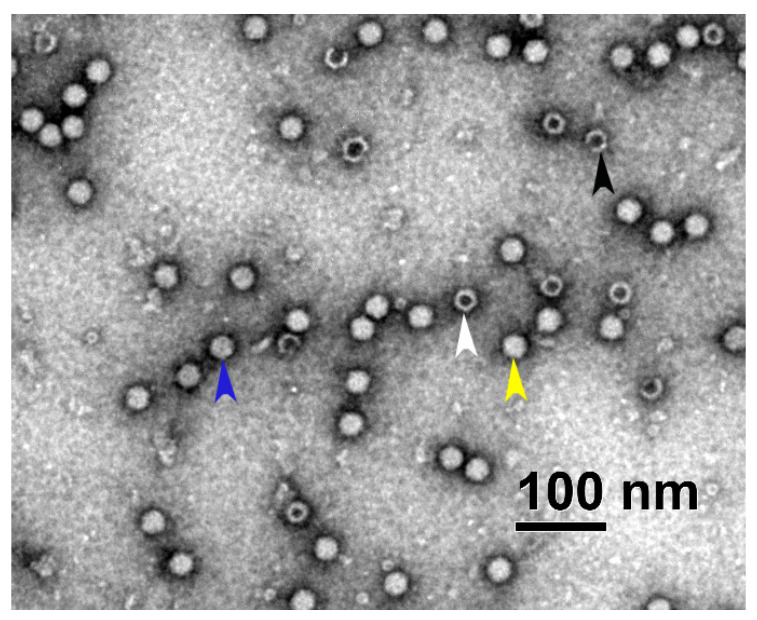
Representative part of the scAAV heavy fraction micrograph taken with a TEM Philips CM 100 showing different viral particles (blue = partially filled particle, black = damaged particle, white = empty particle and yellow = full viral particle). The full size micrograph can be found in the Supplementary Materials (Figure S2).

To assess the presence of different viral particles in each fraction using TEM, the samples were placed in parallel on two sets of cooper grids, GD+ and GD-. Four different types of viral particles (empty, full, partially filled, and damaged) were easily distinguished, as presented in the micrographs (Figure 1).

**Figure 2:**
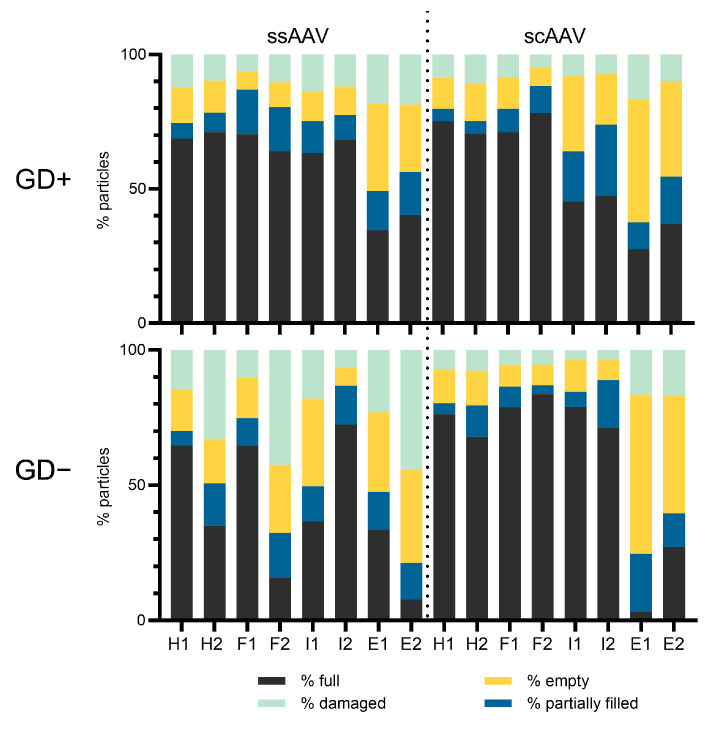
Distributions of the full, partially filled, empty and damaged viral particles using transmission electron microscopy (TEM). Each sample was simultaneously studied on glow-discharged grids (GD+) (1st panel) and on untreated grids (GD−) (2nd panel). The % of each viral particle was determined based on observation of 2000 viral particles separately for two technical replicates of both viral vectors studied. The number (1 or 2) after the fraction name (H = heavy, F = full, I = intermediate, E = empty) represents the technical replicate.

Regardless of the position in the CsCl gradient and the viral vector tested, all the fractions contained all four types of viral particles (Figure 2). Viral particle distribution analysis showed higher variability between the GD+ and GD− grids for the ssAAV samples as well as for the intermediate and empty fractions of scAAV (Figure 2). Furthermore, the repeatability of the results obtained on the GD− grids was lower compared to the GD+ grids (Figure 2); thus, this type of grid was determined to not be suitable for analysis of AAV particles and only the results acquired on GD+ grids were compared to other methods. Representative micrographs of each fraction can be found in the Supplementary Materials (Figures S3 and S4).

A similar percentage of full viral particles was observed in all the technical replicates of the heavy and full fractions for both vector types (68.8% and 71.2% for heavy ssAAV; 70.4% and 63.9% for full ssAAV; 75.1% and 70.5% for heavy scAAV; 71.0% and 78.3% for full scAAV). The percentage of full viral particles was similarly high in the intermediate fraction of ssAAV (63.6% and 68.4%), but noticeably lower in the intermediate fraction of scAAV (45.4% and 47.8%), where partially filled viral particles were more abundant (18.6% and 26.3%) compared to the other studied fractions. As expected, the highest percentages of empty as well as damaged viral particles were observed in the empty fraction for both viral vectors studied (sum of empty and damaged particles for ssAAV was 50.8% and 43.6% and 62.5% and 45.4% for scAAV). Interestingly, the % of partially filled particles was the lowest in all the heavy fractions studied (around 5%).

**Figure 3:**
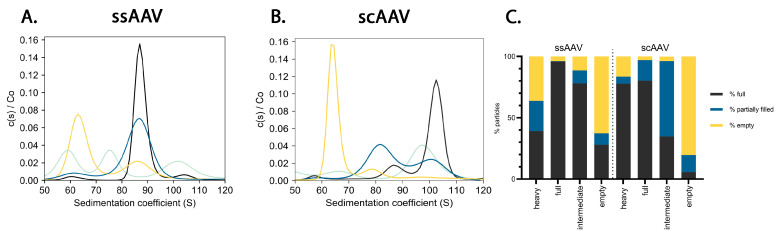
Distribution of the full, partially filled, and empty particles determined using analytical ultracentrifugation (AUC). (**A**,**B**) Representation of the normalized results from the analytical ultracentrifugation of the AAV fractions ((**A**) ssAAV and (**B**) scAAV) (light blue—heavy fraction, black—full fraction, dark blue—intermediate fraction, yellow—empty fraction); both technical replicates of each fraction were combined due to low sample volume. (**C**) Calculated distribution of the particles.

Additionally, the presence of different viral particles in all the fractions was assessed using AUC (Figure 3). Empty capsids were predominantly present in the empty fractions with a sedimentation coefficient around 65 S. A minor peak with this coefficient was also observed in both heavy fractions. Partially filled ssAAV capsids (sedimentation coefficients 70 and 80S) were observed in the heavy ssAAV fraction. Partially filled scAAV capsids (sedimentation coefficients between 80 and 90 S) were predominantly present in the intermediate scAAV fraction and minor peaks were also observed in the empty and full scAAV fractions. Full ssAAV particles with the sedimentation coefficient of 86S were predominantly observed in the full and intermediate ssAAV fractions. A minor peak was also observed in the empty fraction. Full scAAV particles with a sedimentation coefficient of 105 S were observed mainly in the full scAAV fraction and a few of them were also present in the intermediate scAAV fraction. The heavy scAAV fraction contained particles with the sedimentation coefficient around 97 S. The analysis also revealed the presence of macro-molecules and particles larger than full capsids (Table S1), but they were not considered for the full to empty ratio calculation (Figure 3 and Table S2).

### 3.3. Complexity of Viral Particle Content Was Confirmed by Several Different and Orthogonal Approaches

Although TEM and AUC are excellent methods for determining the presence of various viral particles, they have many drawbacks (e.g., long turnaround time, low throughput, large volume of samples and challenging micrograph analysis [7]). Subsequently, these disadvantages resulted in the development of more accessible analytical approaches that compare the viral particle/protein quantities to the estimated vector genome/DNA levels, enabling the estimation of the capsid content or the % of full particles.

As an alternative to TEM and AUC, SEC-MALS was used first. DLS was used prior to the SEC-MALS analysis to quickly determine the particle concentration as well as the presence of aggregates. The hydrodynamic radius was estimated to be between 9 and 16 nm, and the respective hydrodynamic diameter was between 18 and 36 nm, which corresponded to AAVs (Table 3). Since all the fractions are of the same serotype, the low hydrodynamic diameter observed in the heavy ssAAV fraction is mostly due to the different sample viscosity, which could also lead to overestimation of the particle concentration. The particle concentrations ranged from 1.19 × 10^12^ to 3.98 × 10^12^ particles/mL for ssAAV and 2.67 × 10^12^ to 2.11 × 10^13^ particles/mL for scAAV. All the samples contained aggregates to different extents (Figure S5). Although large aggregates cause a strong LS intensity, their mass fraction is mostly below 4% compared to the total mass and their number is generally below 0.1% compared to the total amount of particles.

Next, the presence of viral particles and their potential aggregates were investigated using SEC-MALS. We were able to distinguish between the aggregates (elution time around 6 min), AAV monomers (elution time around 8–9 m) and small UV-active species in all the fractions (Figures S6 and S7). We were not able to determine the concentration of dimers in our samples, since the aggregates and dimer peaks overlapped. Simultaneous measurements of the light scattering as well as the UV signals enable SEC-MALS to determine the total molar mass, molar mass of proteins as well as molar mass of nucleic acids of the samples at the given elution time. These measurements were used to determine the full to total ratio shown in the Table 4. The high SD values observed in the ssAAV heavy fraction were obtained due to the high viscosity of the sample. A similar effect was also observed when this sample was examined with DLS.

**Figure 4:**
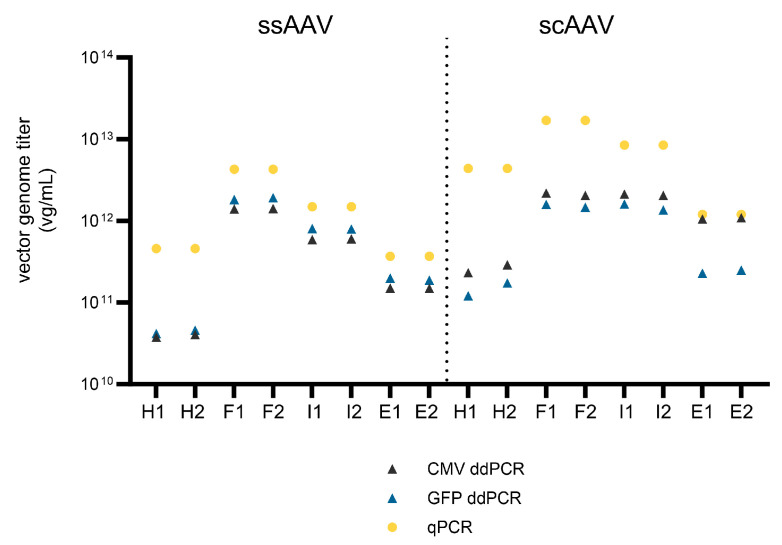
Evaluation of the vector genome titer using qPCR and ddPCR (CMV and GFP assays). The number (1 or 2) after the fraction name (H = heavy, F = full, I = intermediate, E = empty) represents the technical replicate.

In addition to SEC-MALS, the combination of capsid ELISA and ddPCR/qPCR was used to assess the % of full viral particles. First, the viral genome titer was evaluated in parallel by qPCR and ddPCR. Overall, the results obtained with qPCR were higher than those obtained with ddPCR and ranged from 3.7 × 10^11^ to 1.7 × 10^13^ vg/mL with qPCR and 3.8 × 10^10^ to 2.2 × 10^12^ vg/mL with ddPCR (Figure 4). As expected, the highest number of vector genomes was observed in the full fraction for both viral vectors, but the amount of vector genomes was also comparable in the scAAV intermediate fraction. In addition, the heavy and empty fractions also contained more than 3.8 × 10^10^ vg/mL, as determined by ddPCR.

The variability between qPCR and two ddPCR assays targeting different parts of the viral vector genome was assessed in terms of the fold differences in the defined vector genome titer (Table 5). The highest variability between the qPCR and ddPCR results (up to 12.2× for ssAAV and up to 36.2× for scAAV) was observed in the heavy fractions (technical replicate 1) and decreased toward the empty fractions. To assess the importance of selecting the correct genome targets in the ddPCR protocol, we evaluated the vector genome titer using two different ddPCR assays, one targeting the CMV promoter and the other targeting the GFP gene. Interestingly, a higher vector genome titer was defined with the GFP assay when the ssAAV samples were tested and with the CMV assay when the scAAV samples were tested.

**Figure 5:**
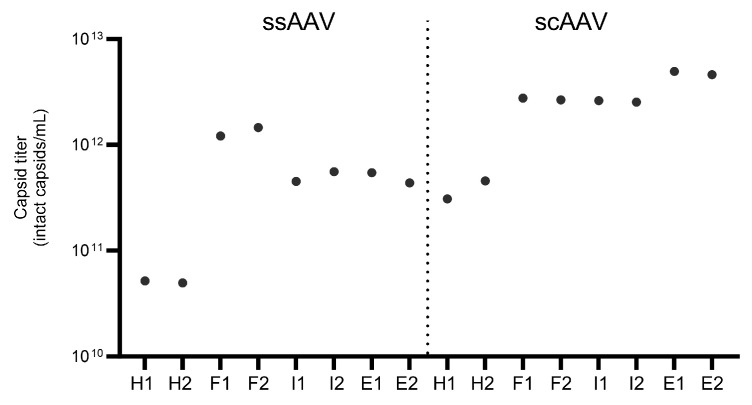
Concentration of intact viral particles defined with ELISA. The number (1 or 2) after the fraction name (H = heavy, F = full, I = intermediate, E = empty) represents the technical replicate.

The concentrations of intact viral particles were determined by ELISA (Figure 5) and ranged from 4.9 × 10^10^ to 5.0 × 10^12^ viral capsids/mL and were thus slightly lower than when determined with DLS (Table 3). The number of capsids was higher in the scAAV sample, as was also observed in the TEM micrographs (Figures S3 and S4), where the number of capsids per micrograph was higher. As expected, the highest number of capsids was observed in the full fraction of ssAAV, but the highest number of intact scAAV capsids was observed in the empty fraction.

**Figure 6:**
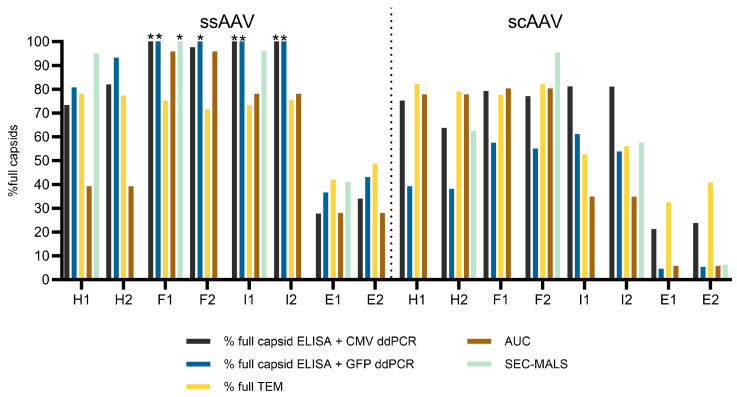
The % of full viral capsids present in each fraction evaluated by 5 approaches (TEM, combination of capsid ELISA and 2 different ddPCR assays, AUC, and SEC-MALS). * = the defined % of full viral particles was higher than 100, meaning that the number of capsids was lower than the amount of vector genomes determined in those samples. The number (1 or 2) after the fraction name (H = heavy, F = full, I = intermediate, E = empty) represents the technical replicate. Due to volume constraints, both technical replicates were combined for AUC and therefore both technical replicates have the same value represented in the graph.

Due to the variability of the genome titers defined either with GFP or with CMV ddPCR assay, we have calculated the % of full viral particles using both results and compared them with results obtained with TEM, AUC, and SEC-MALS (Figure 6). The alternative approaches revealed a higher % of full viral particles across all the ssAAV fractions, except for the empty fractions, where the results aligned with those observed with TEM. Notably, higher discrepancies between the alternative and traditional methods emerged for the scAAV fractions. Particularly, the % of full viral particles was greater when capsid ELISA was coupled with CMV ddPCR assay, as opposed to GFP ddPCR assay.

### 3.4. Duplex ddPCR Reveals High Levels of Vector Genome Fragmentation

**Figure 7:**
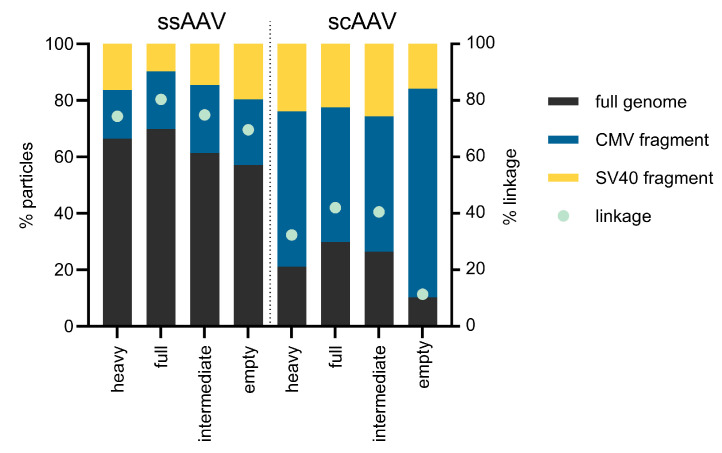
Single-dye duplex ddPCR results showing the presence of full-length genomes as well as the presence of encapsidated genome fragments. Another way of calculating the genome integrity from ddPCR data, i.e., linkage, is also presented. The analysis was performed on one batch of ssAAV and one batch of scAAV fractions.

To understand the fold differences obtained in the two ddPCR assays (Table 5), we developed a single-dye duplex ddPCR for the simultaneous detection of two targets (CMV and SV40) at each end of the vector genome (Figure S1). After converting the number of droplets in each group into the concentration according to the Poisson distribution, we determined that the full fractions contained the highest % of full genomes, followed by the intermediate and heavy fractions for both viral vectors (Figure 7). The lowest amount of full viral genome was present in the empty fractions. Interestingly, the % of full genomes was higher in the ssAAV samples (around 60% in all fractions) compared to the scAAV samples, where the % full was around 30% in the full fractions and even lower in the other fractions. As confirmation of the results, the integrity was calculated using another approach, linkage. When the % of linkage was calculated for all the fractions, a similar pattern was observed (i.e., the highest % linkage was observed in full fractions and the lowest in empty fractions). Interestingly, the % linkage showed a somewhat higher % of full vector genomes in both vectors studied (around 70% for ssAAV and around 10 to 40% for scAAV).

### 3.5. Host Cell Residuals and Viral Particle Aggregates Were Observed in All Fractions

**Figure 8:**
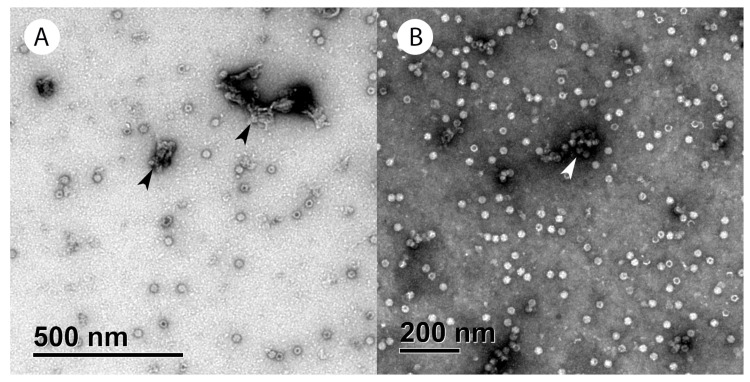
Host cell impurities evaluated with TEM. (**A**) Parts of the host cells (marked with black arrow). (**B**) Huge viral particles aggregated (marked with white arrow). Both types of impurities were observed mostly, but not exclusively, in the intermediate and empty fractions.

In parallel to the analysis of the viral particles from the CsCl gradient, the presence of contaminants (viral aggregates, residual host cell proteins and DNA) was investigated using TEM and molecular methods (ELISA, qPCR and MADLS). In all the fractions studied, host cell impurities and aggregates were directly observed with TEM (Figure 8) but appeared more frequent (no quantitative data available) in the intermediate and empty fractions, i.e., higher in the gradient.

**Figure 9:**
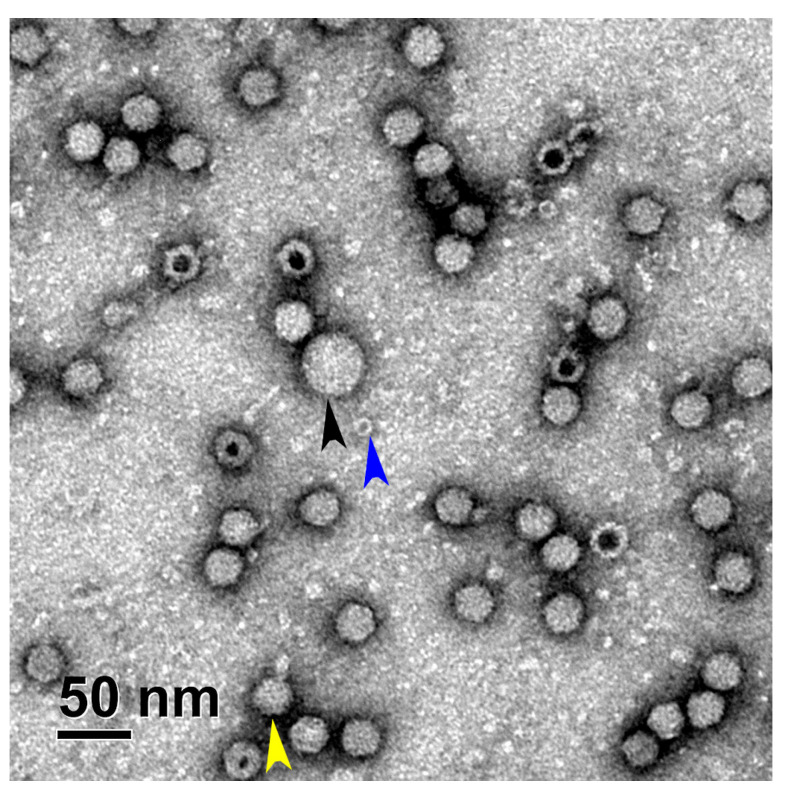
In-depth analysis of the TEM micrographs showed the presence of different contaminants (e.g., host cell proteins—blue arrow, bigger icosahedral viral particle—black arrow, and potentially AAV viral particles with viral Rep protein complexes attached to them—yellow arrow).

In addition to the larger host cell contaminants, smaller protein-like structures can also be observed in the TEM micrographs. For example, small ring-like protein structures in the size of around 12 nm (Figure 9, blue arrow). Similar protein impurities have previously been identified as ferritin in preparations derived from ultracentrifugation gradient [37,38]. However, these impurities should undergo further examination, such as mass spectrometry, to exclude that they are not 20S proteosomes, which were observed in another study [8]. Additionally, larger icosahedral viral particles were observed alongside the rAAV particle (Figure 9, black arrow). Interestingly, we observed many AAV viral particles with a short protein tail attached to the surface of the capsid, potentially representing the Rep protein complexes that transfer DNA molecules within the capsid (Figure 9, yellow arrow).

**Figure 10:**
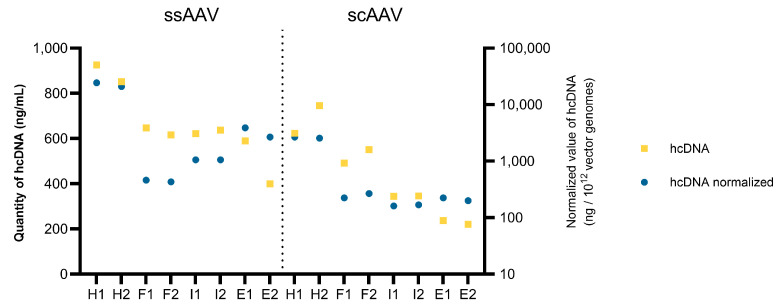
Average and normalized values of residual host cell DNA in all the fractions for both viral vectors. The number (1 or 2) after the fraction name (H = heavy, F = full, I = intermediate, E = empty) represents the technical replicate.

Next, the presence of host cell DNA was assessed using commercially available kits for DNA isolation and quantification based on qPCR. The amount of host cell DNA de-creased from the heavy fraction to the empty fraction for both viral vectors examined (Figure 10, yellow squares). Overall, the amount of host cell DNA was higher in the ssAAV samples. To compare the amount of unwanted DNA between the fractions, considering the number of vector genomes, the results were normalized on 1 × 10^12^ vector genomes. After normalization, the differences between both heavy fractions and the other fractions were even more pronounced (Figure 10, blue circles), whereas for the other fractions, the normalization effect differed (e.g., for the full fraction, the quantity dropped when compared to the other fractions).

**Figure 11:**
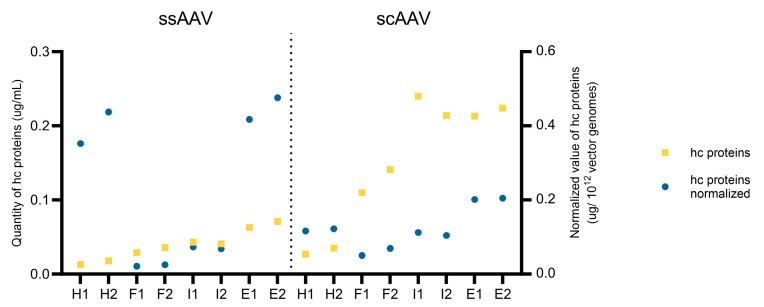
Average and normalized values of the host cell proteins in each fraction for both viral vectors. The number (1 or 2) after the fraction name (H = heavy, F = full, I = intermediate, E = empty) represents the technical replicate.

Another commercially available ELISA kit was used to evaluate the presence of host cell proteins. Compared to the host cell DNA, the amount of host cell proteins increased from the heavy to the empty fractions (Figure 11, yellow squares). Overall, the amount of host cell proteins was higher in the scAAV samples. As with the host cell DNA, the results were normalized based on the vector genomes in the sample (Figure 11, blue circles), resulting in higher variability between the fractions. The highest normalized amounts of host cell proteins were observed in the heavy and empty fractions for both viral vectors.

**Figure 12:**
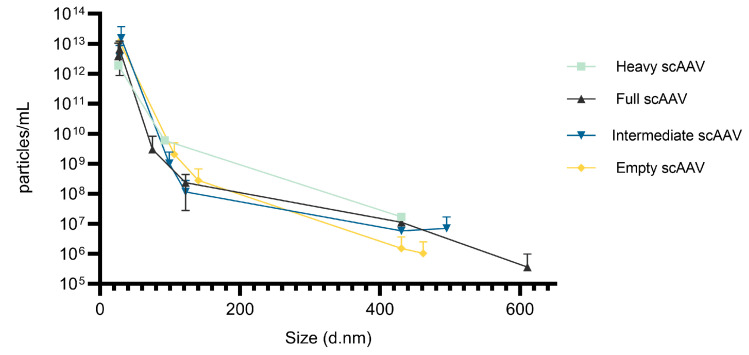
The presence of viral particles, where smaller and larger aggregates were observed with MADLS in one technical replicate of each scAAV fraction.

Next, the presence of viral particles and their potential aggregates in one batch of scAAV samples was additionally investigated using MADLS (Figure 12). Using MADLS, we were able to distinguish between single viral particles (first peak at an average diameter size of 30 nm), smaller AAV aggregates (second peak at an average diameter size of 120 nm), and larger aggregates (third peak at an average diameter size of 430 nm) (Figure 12 and Figure S8) in all four fractions studied.

We observed the highest number of intact particles in the intermediate and empty fraction and the smallest number in the heavy fraction. In contrast, most of the smaller and larger aggregates were observed in the heavy fraction and the least in the empty fraction. The intermediate and full fractions had similar amounts of aggregates, except for the additional larger aggregates in the full fraction in a diameter size of 610 nm, which were not detected in any other sample.

### 3.6. High-Throughput Sequencing (HTS) Holds Tremendous Potential for the Characterization of Unwanted DNA Impurities in rAAVs

HTS was used to evaluate the content of all the nucleic acids present in the sample and to make a relative assessment of the vector genome content compared to impurities. To determine the nucleic acid profile of the capsid content, two different treatments were prepared and compared. One part of the sample was left untreated, another part was treated with DNase I to remove all the free nucleic acids and allow sequencing of only the encapsidated content. In addition, second DNA strand synthesis was performed on the portion of samples to also allow comparison of the nucleic acid profile in the form of a single or double strand.

**Figure 13:**
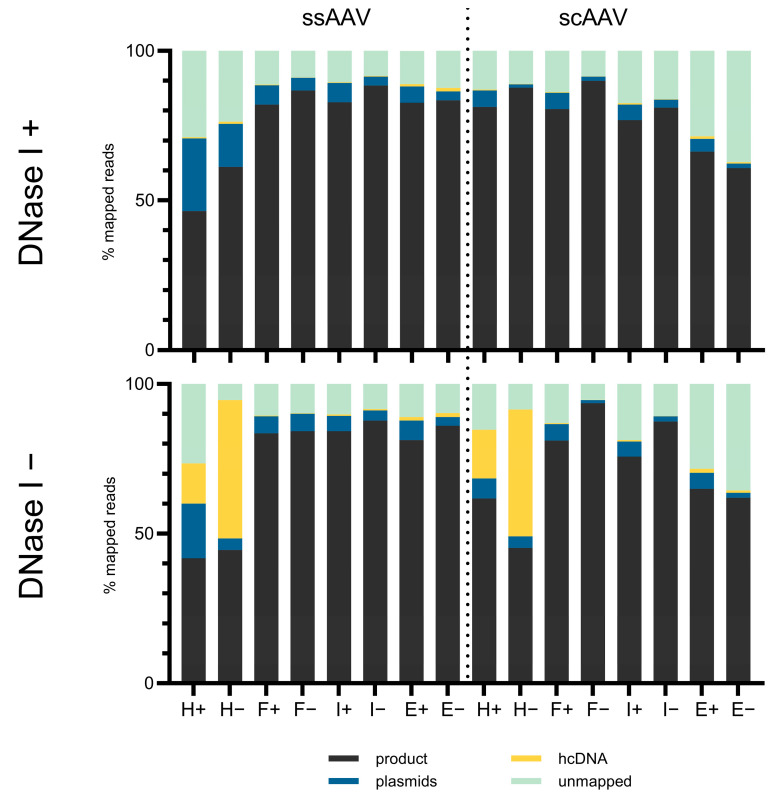
Read mapping results expressed as the % of total Illumina sequencing reads for each DNase I treated sample and DNase I untreated samples. Additionally, 2nd DNA strand synthesis was performed (+) or not (−). H = heavy, F = full, I = intermediate and E = empty fraction. The product represents the % of total reads mapping to the rAAV genome from ITR to ITR, the plasmids represent the % of total reads mapping to any of the plasmids used in the AAV production, and the hcDNA represents the % of total reads mapping to the human genome. Both technical replicates of each fraction were combined due to the low sample volume.

We have detected viral vector genome, plasmid DNA and hcDNA contamination in all the samples (Figure 13). Most of the reads mapped to the expected viral vector genome (i.e., the DNA sequence between the ITRs on the GFP plasmid), but we have also found a few significant differences between the sample pretreatments. When comparing the samples with or without DNase I treatment, we observed a significant difference in the heavy fraction, where hcDNA was present in a much higher concentration in the samples without DNase I treatment. This indicates that the majority of the detected hcDNA was present outside the capsids. In addition, second DNA strand synthesis resulted in a lower relative hcDNA content in these samples, suggesting that the hcDNA was already present in the form of dsDNA, as second DNA strand synthesis only increased the number of vector genomes present in the form of ssDNA, reducing the relative proportion of hcDNA. There were no other significant differences between the samples of the different fractions, as the profiles of the nucleic acids were comparable. There was a proportion of unmapped reads in all the samples, some of which were not mapped due to the stringent mapping conditions, although some of these reads could also be chimeric reads or nucleic acids of other origin that are expected to be present as a background in any laboratory preparations (e.g., due to their presence in reagents).

Read mapping to the expected vector genome sequence revealed some differences between the ssAAV and scAAV vectors (Figures S9 and S10). In all the libraries, the ITR regions were poorly sequenced, probably due to the complex secondary structure and the high CG content of these regions. In all the heavy and full samples, the highest coverage of the vector genome was at the regions of the CMV promoter, the chimeric intron, and the beginning of the GFP gene. The beginning of the CMV enhancer and the SV40 poly(A) signal regions had a lower coverage. On the other hand, in the intermediate and empty fractions of scAAV, the region with highest coverage was the end of the CMV enhancer, regardless of the sample pretreatment protocol. Coverage then gradually decreased toward the non-mutated ITR regions. However, different coverage patterns were observed in the differently pretreated intermediate and empty fractions of ssAAV. After second DNA strand synthesis, the coverage was the highest at the beginning of the GFP gene, whereas without second DNA strand synthesis, the highest coverage was at the start of the CMV enhancer or at the SV40 poly(A) signaling region.

## 4. Discussion

We adopted a holistic approach and analyzed the presence of intact bioactive AAV9 particles and contaminants such as undesirable viral particles as well as DNA molecules and host cell proteins using several different analytical methods in the CsCl density gradient fractions. Numerous published articles explore the applicability and correlations between two or more analytical methods for different AAV serotypes [4,6,8,9,10,11,13,23]. However, to the best of our knowledge, no study has simultaneously investigated the comparability of methods for both single-stranded and self-complementary genomes of AAV9.

We prepared four ssAAV and four scAAV fractions by CsCl ultracentrifugation: heavy, full, intermediate, and empty, and analyzed their infectivity. The observed infectivity was comparable for all the fractions, with the heavy ssAAV fraction being the least infectious among the ssAAV fractions (14.6% of GFP positive cells) and the intermediate scAAV fraction being at least infectious among the scAAV fractions (13.9% of GFP positive cells).

Unlike our relatively consistent infectivity across fractions, other studies have showed greater variability in GFP expression among AAV8 fractions (ssAAV genomes) from CsCl ultracentrifugation gradients [39] or among AAV9 STRV5 fractions (scAAV genomes) using live-cell imaging and GFP ELISA assays [23]. Between-study comparisons should be performed with caution, as there is a discrepancy between serotypes and methods for AAV infectivity assessment (GFP expression determined with flow cytometry vs. live-cell imaging and GFP ELISA assay). Furthermore, an additional chromatography step prior CsCl ultracentrifugation [23], or improved ultracentrifugation protocol could lead to more efficient viral particle separation. This, in turn, might result in higher variability in the transduction efficiencies between fractions. Likewise, previous studies have highlighted discrepancies in the methods used to determine rAAV infectivity in vitro [40]. Additionally, infectivity levels vary even among the same serotype or its variants, depending on the cell type being used in the study [41]. Several studies used AAV9 for infecting different types of cells; however, a focus was placed on transgene delivery and not on the infectivity levels [42,43,44].

The relatively similar results of the infectivity assay caught our attention and served as the starting point for the presented study, as they called for explanation. Thus, we aimed to characterize the fractions using different, in some cases orthogonal, methods.

First, we attempted to assess the presence of different viral particles (full, partially filled, empty, and damaged) using TEM, AUC, SEC-MALS as well as with a combination of vector genome titer determination by either qPCR or ddPCR and capsid ELISA. In addition, the presence of host cell proteins, host cell DNA and aggregates was evaluated with ELISA, qPCR, TEM, MADLS and HTS.

When the fractions were analyzed by TEM, all the samples were simultaneously put on two types of copper grids, GD+ and GD−. Other groups have reported using uranyl acetate as a negative stain on either GD+ grids [8,11,14,37,45,46] or the glow-discharge status was not precisely defined [9]. In our experience with AAV samples, the spread across the grid is highly sample-dependent, so we routinely test samples on both grid types. Interestingly, the % of empty and damaged particles is higher on the GD− grids, suggesting that those particles might have different affinity for the surface due to their status and content. Due to the uneven spread of the ssAAV fractions on the GD− grids, we observed different outcomes when comparing technical replicates. Consequently, we chose to include only the GD+ results when TEM was compared to other orthogonal methods.

Using TEM, we were able to detect the presence of full, empty, partially filled, and damaged viral particles in all the ssAAV and scAAV fractions, suggesting that the separation of particles with two runs of CsCl ultracentrifugation was not optimal. Others have reported the presence of full viral particles in supposedly empty AAV samples as well as the presence of empty particles in full samples with an improved purification protocol in TEM analysis [8,11,16], but at a much lower level than what we observed. The % of full viral particles was lower in the empty fractions compared to the full or intermediate, but remarkably, this did not impact the infectivity of those fractions. When we compared the TEM results to other orthogonal methods (Figure 6), we found that TEM slightly underestimates the percentage of full particles in all the ssAAV fractions, except for the empty fraction, but overestimates the percentage of full particles in the empty scAAV sample; however, the challenge of accurately defining AAV particles as full using TEM has already been described [4,6,8,11]. In order to evaluate the presence of partially filled viral particles, the samples were additionally analyzed by AUC, which is the only method that can detect partially filled as well as overfilled subpopulations of particles [6,8,10]. AUC revealed that the scAAV fractions have a distinctive centrifugation profile and each fraction contains predominantly one type of particle. However, full and partially filled viral particles were also observed in the empty fraction and empty particles were also observed in the full and intermediate fractions. The centrifugation profiles of the ssAAV fractions were slightly different to the scAAV. The full and empty fractions predominantly contained either full or empty particles similarly to the scAAV fractions. However, the partially filled particles were predominantly present in the heavy ssAAV fraction and not in the intermediate fraction, where a lot of full particles were present. A similar distribution of scAAV particles has been described by others [10]; however, they have used CDMS and not TEM as an alternative method. The discrepancy between the TEM and AUC results was observed before [8,47], although another study reported that results from TEM and AUC to be comparable [4].

In addition to TEM and AUC, the % of full viral vectors was also evaluated with SEC-MALS and a combination of ddPCR and ELISA (vg/vp approach). Both approaches involve comparing the particle/protein quantities to the estimated vector genome/DNA amounts, allowing us to estimate the capsid content or % full particles. However, it is crucial to recognize that employing this approach increases the risk of inaccuracies, as both components (e.g., proteins and DNA content) can be inaccurately identified.

Inconsistency in the % of full viral particles obtained with the SEC-MALS, AUC, or vg/vp approach has previously been documented, particularly in the context of samples predominantly containing filled particles and when qPCR was utilized instead of ddPCR [6,11]. In contrast to TEM, the SEC-MALS as well as ddPCR/ELISA results show that the ssAAV particles from the heavy, full, and intermediate fractions appear to be completely filled with vector genome. Considering the higher amount of vector genomes relative to capsids determined by dPCR/ELISA and the increased molar mass of nucleic acids determined by SEC-MALS, we hypothesize that multiple fragments of nucleic acids may be packed in some particles. The genome length of our ssAAV construct was 2.4 kb; thus, there is a possibility that capsids also packed shorter fragments or even up to two full genomes. However, the AUC results indicate that this is not happening in most of the capsids. There is a difference between the sedimentation coefficients of the full particle populations of ssAAV and scAAV for a value of 19 and the partially filled capsids for scAAVs are in the region of the sedimentation coefficient where the full capsids are for the ssAAVs (Figure 3). Thus, the ssAAV capsids are most probably predominantly filled by only one strand of genome. Nevertheless, there were some overfilled capsids detected in the full and heavy fraction of the ssAAV samples (Figure 3). Recent advancements in long-read sequencing have revealed the existence of vector genome dimers, plasmid or host cell DNA chimeras, which may contribute to the overestimation of the DNA content using SEC-MALS [48]. The full/empty ratios determined for scAAV with SEC-MALS were within the expected range, with the highest percentage of full particles observed in the full fractions, followed by the heavy and intermediate fractions. A relatively low percentage of full particles was observed in the scAAV empty fraction, corresponding to the AUC analysis.

Next, our study emphasizes the importance of selecting the right target for ddPCR. Notably, titers not only vary among different ddPCR targets but also compared to qPCR, resulting in a distinct % of full viral particle determination. In the past, both our observations and those of others have highlighted vector genome titer variations [5,14,16,49]. Recently, Wada et al. conducted a study in which they examined this phenomenon using 22 different ddPCR primer sets on ssAAV9 samples in order to detect the whole region of the ssAAV genome in full and empty particles [16]. Interestingly, they demonstrated that titers of transgene could be higher than those of the ITR or promoter region in samples primarily composed of full particles. On the other hand, higher titers of ITR or promoter regions were detected in samples containing predominantly empty capsids. In contrast, we detected a higher titer of GFP target in all the ssAAV samples. Again, like the TEM and infectivity assay, this suggested that the particles may not have been well separated from each other. On the other hand, higher variability in the vector genome titers was observed in the scAAV samples, resulting in underestimation of the % of full viral particles compared to the values obtained by other methods. This suggests that not only partly filled viral particles but also full viral particles may contain shorter parts of the prepared vector genome.

Additionally, we employed single-channel multiplex ddPCR to explore the integrity of the genetic content within the capsid. Our observations revealed significant differences in genome fragmentation between the ssAAV samples and scAAV samples. Approximately 70% of the ssAAV genomes remained intact across all four ssAAV fractions, corresponding to similar infectivity levels, while only 40% were intact in the full and intermediate scAAV fractions. Notably, the vector genomes in the heavy and empty scAAV fractions exhibited even greater fragmentation, corresponding to high variability between the titers determined with CMV and GFP single-target ddPCR. Nevertheless, these results need to be considered with some caution. Due to the short genome length and the possibility that more than one genome/fragment was packaged in the capsid, the integrity results obtained by multiplex ddPCR might not show the completely accurate picture. This approach also cannot be used to precisely evaluate scAAV genome integrity, since CMV as well as GFP targets are duplicated on each side of the mutated ITR region. In other words, double-positive droplets can contain either true full-length genomes or only half-length fragments that contain both targets on one or other side of the mutated ITR region.

Similar to the variability observed in the quantification of nucleic acids, there can also be variations when estimating the quantity of capsids or proteins using the vg/vp approach to determine the % of full viral particles. A lower particle titer was determined by ELISA than by DLS for all the fractions. It has been reported that DLS measurements are generally inaccurate, but the estimated capsid titer should be improved using MADLS [7]. However, additional measurements of the scAAV fractions by MADLS did not improve that. The determined titers were even higher, mostly because of the viscosity of the sample, which affects the MADLS measurements.

MADLS is still an important analytical approach for the characterization of AAV, as it enables the determination of AAV aggregates [13]. In our study, the presence of AAV aggregates was additionally evaluated by TEM, SEC-MALS and also by AUC for the scAAV samples. All the methods showed the presence of aggregates in all the fractions. However, TEM revealed more aggregates in the intermediate and empty fractions, whereas MADLS, SEC-MALS and AUC determined a higher level of aggregates in the heavy fractions. Wright et al. observed that the presence of nucleic acid impurities contributes to the vector aggregation [50], which is in concordance with our HTS as well as hcDNA results, where the heavy fraction of both scAAV and ssAAV contained the highest amount of host cell DNA.

High-throughput sequencing confirmed the results of the other methods, as there was no significant difference in the nucleic acid profile between the samples of different fractions, except for the heavy fraction. Inside viral capsids, a low relative level of plasmid DNA and an even lower level of hcDNA was detected. Similar read distributions were already shown by other groups [23]. Although HTS is not quantitative, it can provide a very good insight into the presence of all kinds of nucleic acid impurities, even adventitious viruses if present. Newer developments in the field of long-read sequencing enable even better insights into the encapsidated fragment populations, as they can theoretically sequence the whole vector genomes and encapsidated fragments [10,48,51,52,53].

## 5. Conclusions

In conclusion, we have demonstrated that a full understanding of AAV vectors still requires combining multiple analytical methods. There is no single method that can provide the most informative results for several parameters at once. The observed infectivity levels of the fractions were in our case unexpectedly similar, which can be explained by the comparable composition of the samples and the high proportion of full infective capsids in all the fractions, most probably due to insufficient viral particle separation. The only major difference between the fractions was the amount of empty particles present in the empty fractions. Different groups in the gene therapy community have reported different results regarding the impact of empty capsids on the transduction ability of full capsids. Our research validates that, at least at the cellular level, empty capsids do not impede the function of full capsids. However, a lingering question remains: Are empty capsids truly devoid of content? Could recombination take place among the fragments within these seemingly empty capsids, resulting in complete vector genomes? Consequently, even empty capsids might serve as full capsids. Additionally, our study proved that comparisons between different studies should always be performed with caution, since the results obtained with the same methods vary between different sample types. Furthermore, our results have highlighted a problematic insight into the level of vector genome fragmentation between ssAAV and scAAV, indicating the need for additional analytical approaches for the assessment of vector genome fragmentation in the future. In addition, understanding whether the additional hairpin structure in scAAV causes the higher level of fragmentation would help us improve vector genomes in the long term.

## Figures and Tables

**Table 1:** Primers and probes used for quantification of the physical titer using ddPCR. In all the ddPCR reactions, forward and reverse primers were used at the final concentration of 900 nmol/L and probes at 250 nmol/L.

Target	Label	DNA Sequence of Oligonucleotide (5′ to 3′)	Reference
CMV ^a^	FP-CMV	GTCAATGGGTGGAGTATTTACGG	[34]
	RP-CMV	GCATTATGCCCAGTACATGACCT	
	P-CMV	FAM-CAAGTGTAT/ZEN/CATATGCCAAGTACGCCCCC-BkFQ	
GFP ^b^	FP_GFP_nib	CAGGAGCGCACCATCTTCTT	This study
	RP_GFP_nib	CGATGCCCTTCAGCTCGAT	
	P_GFP_nib	FAM-ACGGCAACT/ZEN/ACAAGACCCGCGC-BkFQ	
SV40 ^a^	FP_SV40	AGCAATAGCATCACAAATTTCACAA	[34]
	RP_SV40	CCAGACATGATAAGATACATTGATGAGTT	
	P_SV40	FAM-AGCATTTTT/ZEN/TTCACTGCATTCTAGTTGTGGTTTGTC-BkFQ	

^a^ Primers used in the simplex and duplex ddPCR reactions. ^b^ Primers used in only the simplex ddPCR reactions.

**Table 2:** Transduction efficiency results and viral titer as determined by qPCR.

Viral Vector	Fraction	% of GFP Positive Cells	Viral Titer (Copies/mL)
ssAAV	Heavy	14.6	4.6 × 10^11^
	Full	22.0	4.3 × 10^12^
	Intermediate	23.0	1.5 × 10^12^
	Empty	21.8	3.7 × 10^11^
scAAV	Heavy	16.1	4.4 × 10^12^
	Full	21.5	1.7 × 10^13^
	Intermediate	13.9	8.5 × 10^12^
	Empty	17.3	1.2 × 10^12^

**Table 3:** Batch DLS measurements.

Viral Vector	Fraction	Radius (nm)	Hydrodynamic Diameter (nm)	Particle Concentration (Particles/mL)
ssAAV	Heavy	9.1	18.3	3.05 × 10 ^12^
	Full	15.9	31.7	1.19 × 10 ^12^
	Intermediate	14.5	29.0	1.41 × 10 ^12^
	Empty	11.7	23.4	3.98 × 10 ^12^
scAAV	Heavy	11.7	23.4	2.67 × 10 ^12^
	Full	13.7	27.5	4.73 × 10 ^12^
	Intermediate	13.8	27.6	6.57 × 10 ^12^
	Empty	13.3	26.6	2.11 × 10 ^13^

**Table 4:** Results of size-exclusion chromatography coupled with multi-angle light scattering (SEC-MALS) obtained from one batch of ssAAV and scAAV fractions.

Viral Vector	Fraction	Molar Mass Total (kDa)	Molar Mass Protein (kDa)	Molar Mass Nucleic Acid (kDa)	Full to Total Ratio (Vg/Cp)
ssAAV	Heavy	4418	3720	698.1	0.953
	Full	4547	3738	782.2	1.114
	Intermediate	4666	3951	715.1	0.961
	Empty	4208	3906	302.0	0.407
scAAV	Heavy	4575	3728	847.3	0.625
	Full	5006	3711	1296	0.955
	Intermediate	4643	3836	807.4	0.575
	Empty	3803	3718	85.4	0.062

**Table 5:** Fold difference between the vector genome (vg) titers defined using qPCR and two different ddPCR assays.

Viral Vector	Fraction	Technical Replicate	qPCR vs CMV ddPCR	qPCR vs GFP ddPCR	GFP vs CMV ddPCR
ssAAV	Heavy	1	12.2	11.0	0.9
		2	11.4	10.0	0.9
	Full	1	3.1	2.4	0.8
		2	3.0	2.2	0.7
	Intermediate	1	2.5	1.9	0.7
		2	2.5	1.9	0.8
	Empty	1	2.5	1.8	0.8
		2	2.5	2.0	0.8
scAAV	Heavy	1	18.9	36.2	1.9
		2	15.2	25.3	1.7
	Full	1	7.7	10.7	1.4
		2	8.3	11.6	1.4
	Intermediate	1	4.0	5.3	1.3
		2	4.1	6.2	1.5
	Empty	1	1.1	5.3	4.6
		2	1.1	4.8	4.4

## Data Availability

Data are available on request from the authors. Raw sequencing data are available from the NCBI SRA under PRJNA1102173.

## Supplementary Materials

The following supporting information can be downloaded at https://www.mdpi.com/article/10.3390/v16081235/s1, Figure S1: A. Schematic representation of the heavy, full, intermediate, and empty fractions following two consecutive CsCl ultracentrifugation gradient runs. No stuffer DNA was used in the ssAAV vector. B. Genome arrangement of both rAAV vectors studied.; Figure S2: Full-size micrograph of the ssAAV empty fraction.; Figure S3: Representative micrographs of each ssAAV fraction on glow-discarded (GD+, left) and on untreated grids (GD−, right).; Figure S4: Representative micrographs of each scAAV fraction on glow-discarded (GD+, left) and on untreated grids (GD−, right).; Figure S5: Representative DLS intensity particle distribution plots (regularization plots) of each fraction studied.; Figure S6: Double detection SEC-MALS chromatograms of each fraction studied.; Figure S7: MALS measurements.; Figure S8: Particle size distribution of the scAAV fractions as determined by MADLS.; Figure S9: NovaSeq read mapping against the predicted ssAAV genome for each tested fraction.; Figure S10: NovaSeq read mapping against the predicted scAAV genome for each tested fraction. Table S1: AUC results—regions from 25–190 S were included in the calculations.; Table S2: AUC results—only 50–120 S regions were included in the calculations.
