# Supplementary material for: In-Depth Comparison of Adeno-Associated Virus Containing Fractions after CsCl Ultracentrifugation Gradient Separation
Source: Viruses. 2024 Jul 31;16(8):1235. doi: 10.3390/v16081235 (PMC11360810; doi:10.3390/v16081235)
Supplement: Supplementary file 1 [file viruses-16-01235-s001.zip › viruses-3007455-supplementary.pdf]

SUPPLEMENTARY MATERIALS

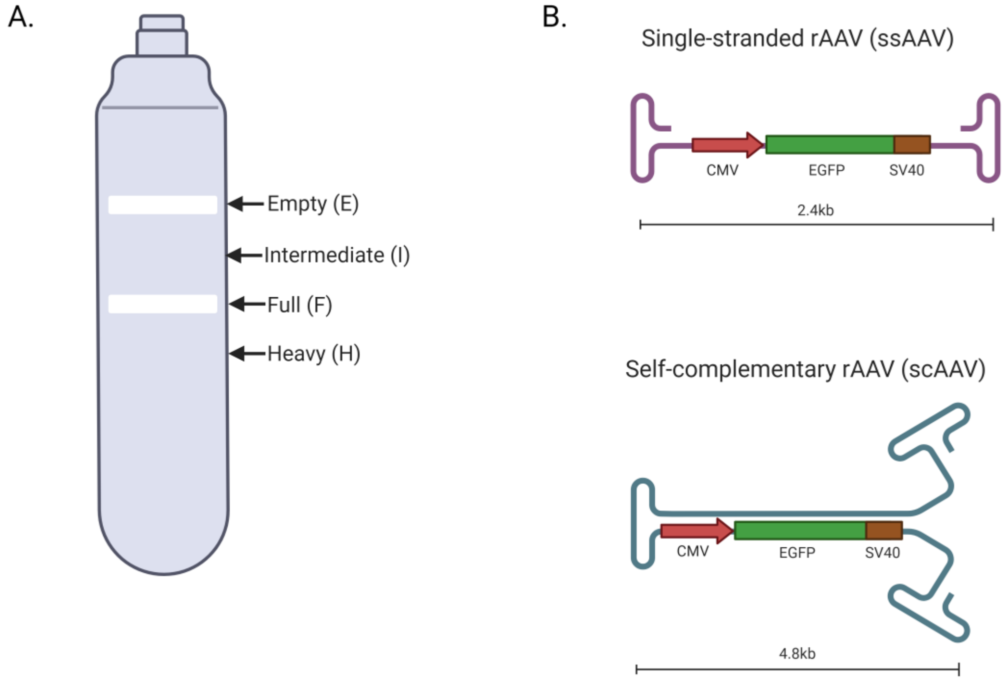

Figure S1 : A. Schematic representation of the heavy, full, intermediate, and empty fractions following two consecutive CsCl ultracentrifugation gradient runs. No stuffer DNA was used in ssAAV vector. B. Genome arrangement of both rAAV vectors studied. Both figures were created with *BioRender.com* (https://app.biorender.com/ (accessed on 27 July 2024).

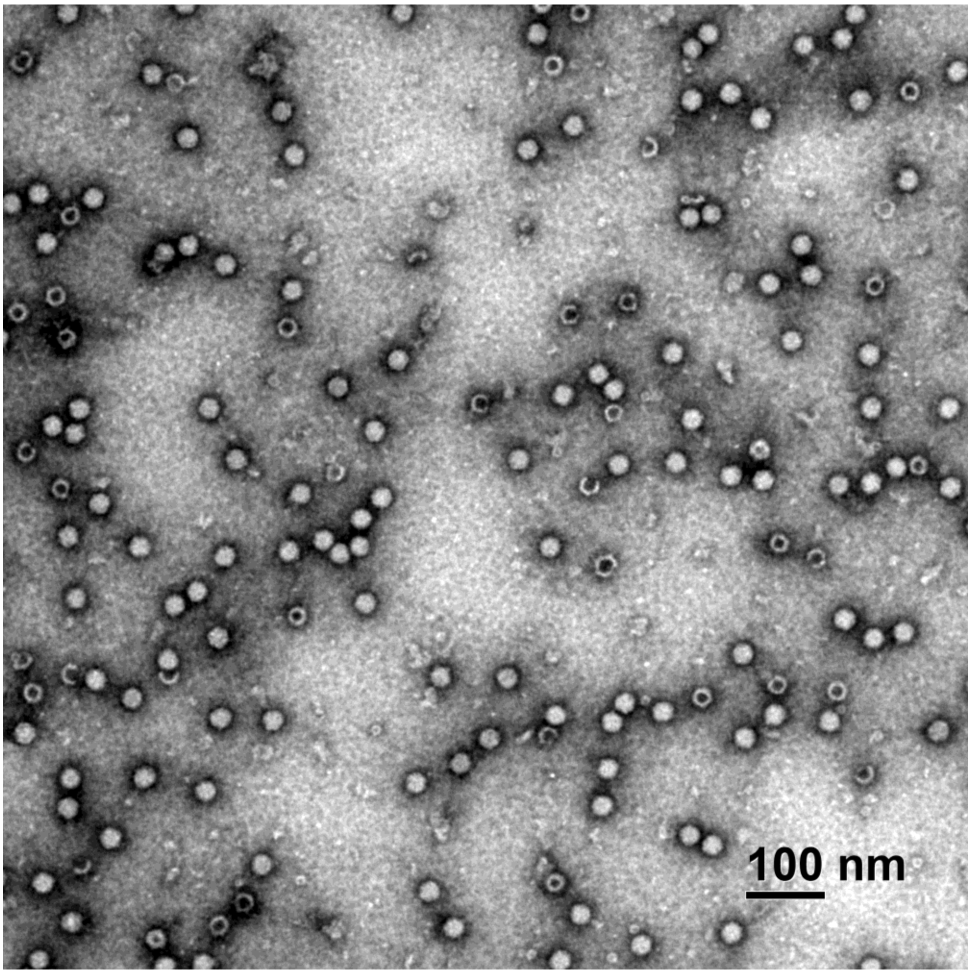

Figure S2: Full-size micrograph of the scAAV heavy fraction.

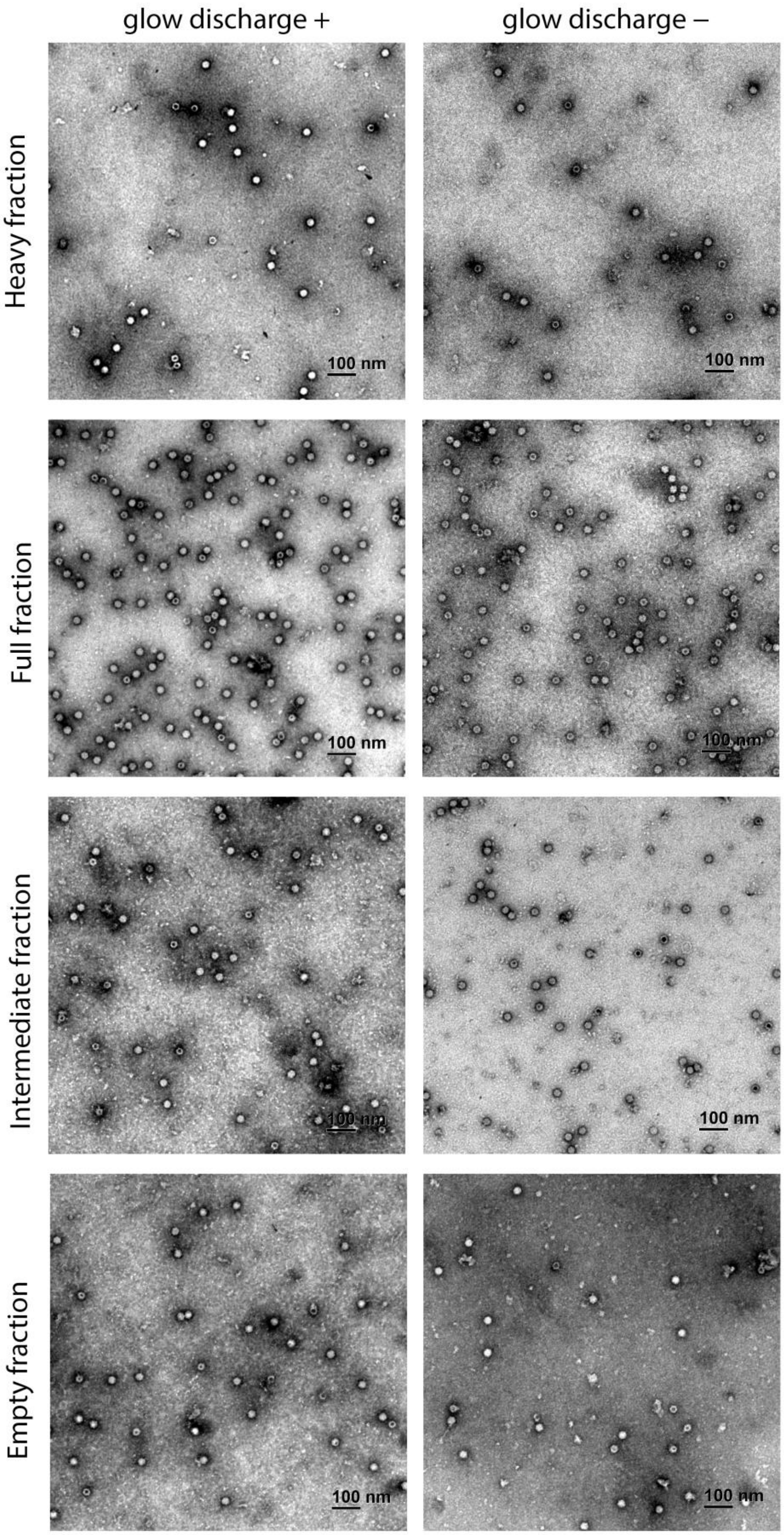

Figure S3: Representative micrographs of each ssAAV fraction on glow-discarded (GD+, left) and on untreated grids (GD−, right).

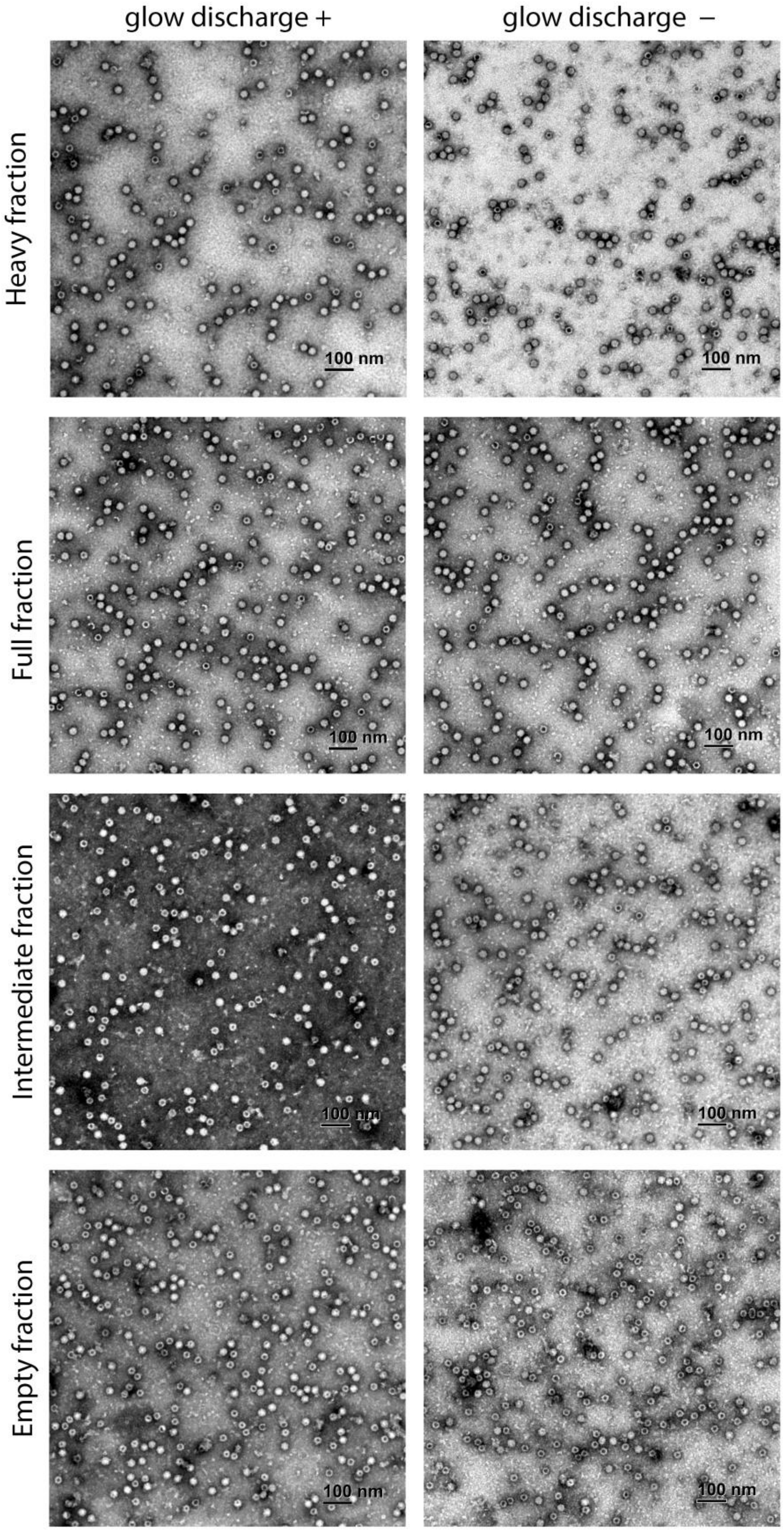

Figure S4: Representative micrographs of each scAAV fraction on glow-discarded (GD+, left) and on untreated grids (GD−, right).

Table S1: AUC results – regions from 25–190 S were included in the calculations.

Viral vector	Fraction	Macromolecule (25–55 S)	Empty capsids (55–70 S)	Partially filled capsids (70–80 S)	Full capsids (80–120 S)	Larger than full capsids (120–190 S)	Very large aggregates (above 190 S)
ssAAV	heavy	27.5%	18.0%	14.0%	22.0%	15.1%	3.4%
	full	1.6%	3.4%	0.2%	90.8%	2.9%	1.1%
	intermediate	7.8%	9.1%	9.2%	69.1%	3.7%	1.1%
	empty	3.5%	57.0%	8.6%	25.4%	2.4%	3.1%
scAAV	heavy	16.9%	10.6%	3.6%	50.0%	1.4%	17.5%
	full	2.1%	2.7%	15.0%	72.6%	6.4%	1.2%
	intermediate	3.7%	3.5%	56.7%	32.3%	2.7%	1.2%
	empty	1.9%	75.6%	13.0%	5.4%	2.4%	1.7%

Table S2: AUC results – only 50–120 S regions were included in the calculations.

Viral vector	Fraction	Empty capsids (55–70 S)	Partially filled capsids (70–80 S)	Full capsids (80–120 S)
ssAAV	heavy	36.1%	24.6%	39.3%
	full	3.8%	0.3%	95.9%
	intermediate	11.3%	10.6%	76.1%
	empty	62.7%	9.3%	28.0%
scAAV	heavy	16.5%	5.6%	77.9%
	full	3.0%	16.6%	80.4%
	intermediate	3.7%	61.3%	34.9%
	empty	80.4%	13.8%	5.8%

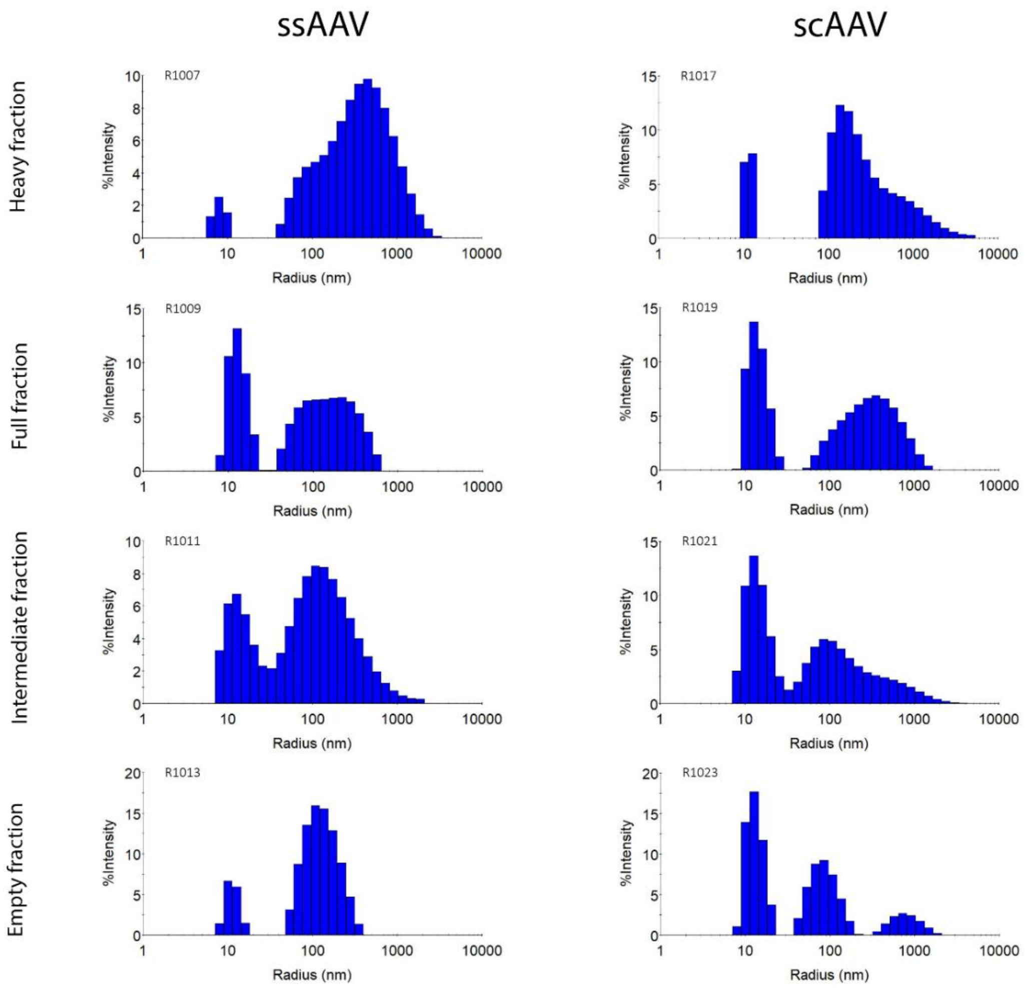

Figure S5: Representative DLS intensity particle distribution plots (regularization plots) of each fraction studied. All samples contained aggregates (2nd peak around 100 nm) to different extents.

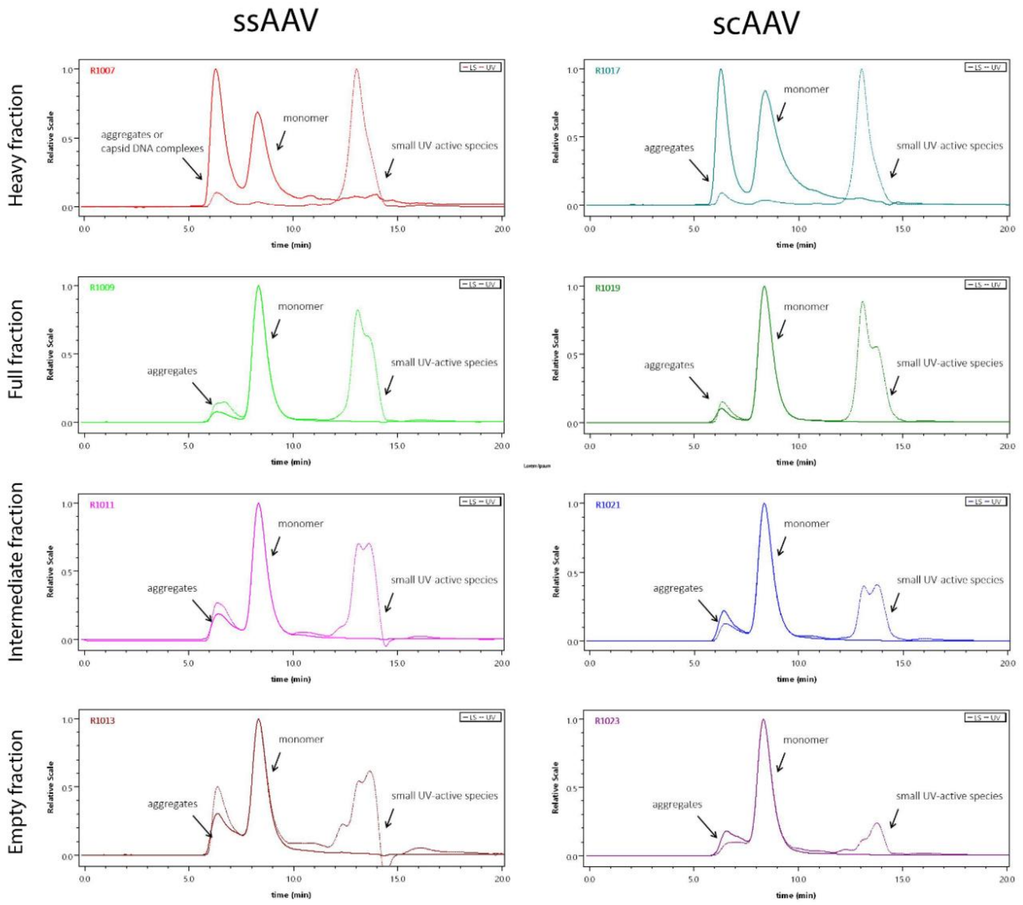

Figure S6: Double detection SEC-MALS chromatograms of each fraction studied. Light scattering (LS) and UV280 were detected simultaneously. Aggregates eluted at 6 min overlapped with dimers and although their concentration is low, they cause strong LS scattering. The highest number of aggregates was observed in heavy fractions. Small UV active species were detected in all samples.

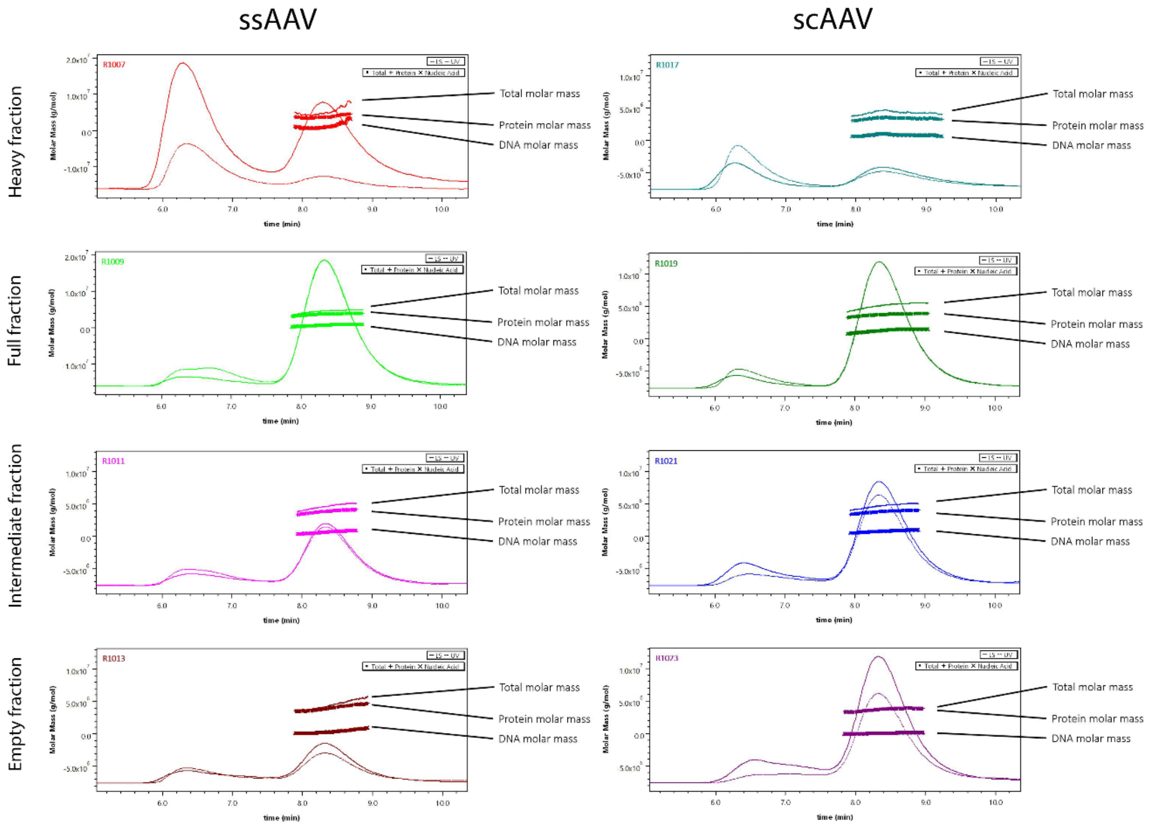

Figure S7: MALS measurements. Simultaneous measurement of UV absorbance and differential refractive index (dRI) during MALS analysis enables the precise determination of total molar mass, protein molar mass and DNA molar mass, which are later used to determine the amount of full viral particles.

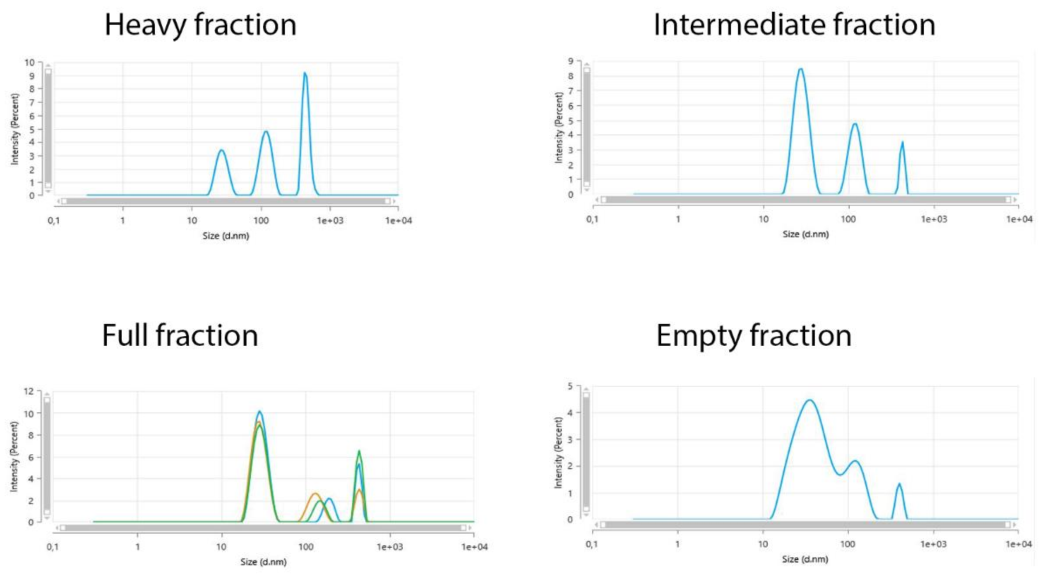

Figure S8: Particle size distribution of the scAAV fractions as determined by MADLS.

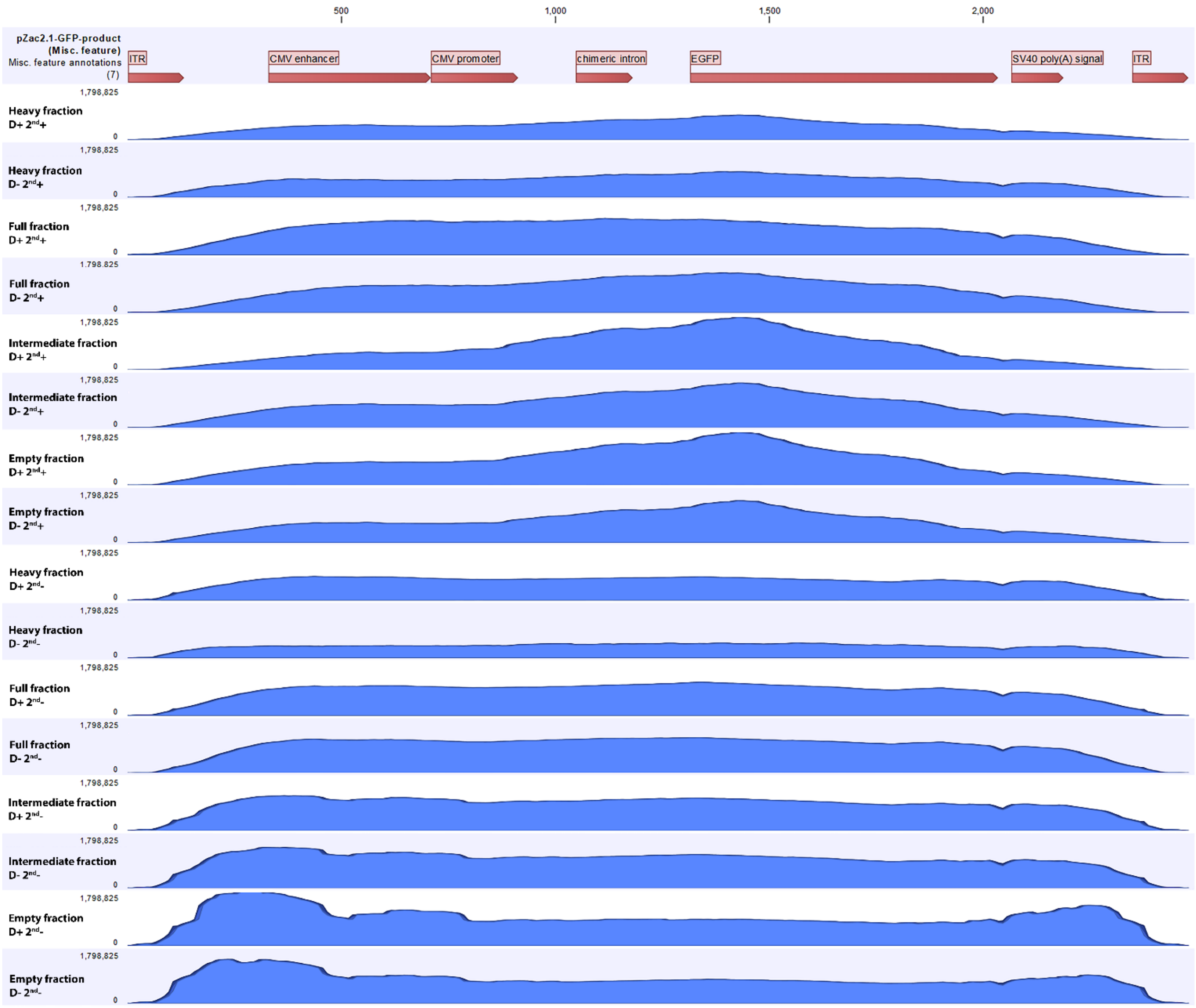

Figure S9: NovaSeq read mapping against the predicted ssAAV genome for each tested fraction. Each fraction underwent sequencing four times, as we employed four different pretreatments: with or without Dnase I (D+ or D−) and with or without second DNA strand synthesis (2nd+ or 2nd−).

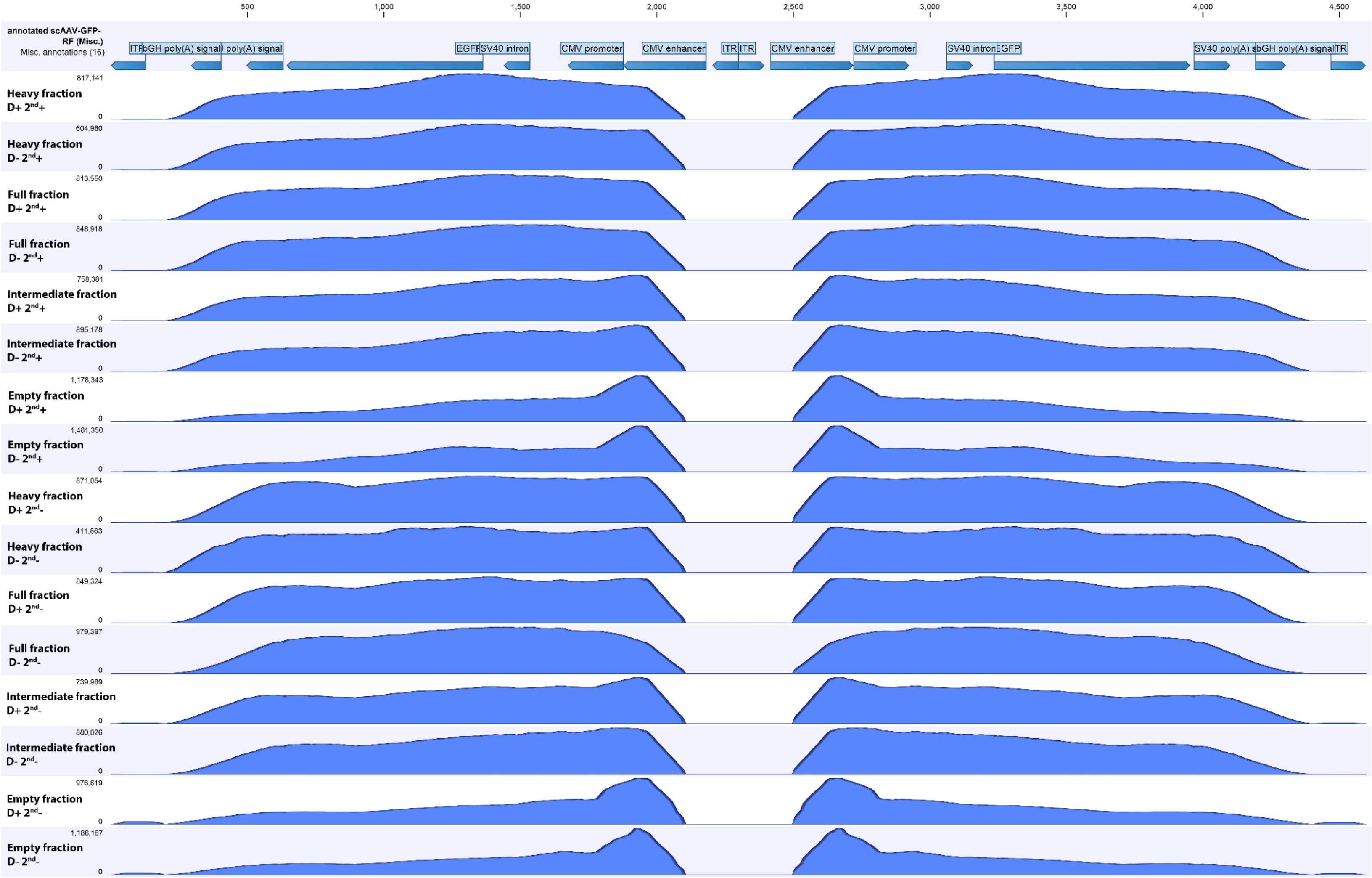

Figure S10: NovaSeq read mapping against the predicted scAAV genome for each tested fraction. Each fraction underwent sequencing four times, as we employed four different pretreatments: with or without Dnase I (D+ or D−) and with or without second DNA strand synthesis (2nd+ or 2nd−)
